# A mixed methods systematic literature review of barriers and facilitators to help-seeking among women with stigmatised pelvic health symptoms

**DOI:** 10.1186/s12905-024-03063-6

**Published:** 2024-04-03

**Authors:** Clare Jouanny, Purva Abhyankar, Margaret Maxwell

**Affiliations:** 1 Faculty of Health Sciences and Sport, University of Stirling, Stirling, Scotland; 2 Department of Psychology, University of Stirling, Stirling, Scotland; 3 The Nursing, Midwifery and Allied Health Professions Research Unit, University of Stirling, Stirling, Scotland

**Keywords:** Pelvic symptoms, Help-seeking, Healthcare, Stigma, Common-sense model

## Abstract

**Background:**

Women’s pelvic health is a globally important subject, included in international and United Kingdom health policies, emphasising the importance of improving information and access to pelvic health services. Consequences of pelvic symptoms are intimate, personal, and varied, often causing embarrassment and shame, affecting women’s quality of life and wellbeing.

**AIM:**

To understand the experience of seeking healthcare for stigmatised pelvic health symptoms by synthesising all types of published primary research and mapping the results to behavioural theory, to identify potential targets for intervention.

**Methods:**

Systematic search of MEDLINE, CINAHL, PsycINFO, SocINDEX, PubMED databases, CDSR and CENTRAL registers, from inception to May 2023 for all types of research capturing women’s views and experiences of seeking help with stigmatised urogenital and bowel symptoms. Studies only reporting prevalence, predictors of help-seeking, non-health related help-seeking, or written in languages other than English, German, French, Spanish and Swedish were excluded. Reference checking and forward citation searching for all included studies was performed. A results-based synthesis approach was used to integrate quantitative and qualitative data. Themes were mapped to the Common-Sense model and Candidacy framework. The Mixed Methods Appraisal Tool was used for critical appraisal. Grading of Recommendations Assessment, Development and Evaluation - Confidence in Evidence from Reviews of Qualitative research for assessing certainty of review findings.

**Results:**

86 studies representing over 20,000 women from 24 high income countries were included. Confidence was high that barriers to help-seeking were similar across all study types and pelvic symptoms: stigma, lack of knowledge, women’s perception that clinicians dismissed their symptoms, and associated normalising and deprioritising of low bother symptoms. Supportive clinicians and increased knowledge were key facilitators.

**Conclusions:**

Using the Common-Sense Model to explore women’s help-seeking behaviour with stigmatised pelvic symptoms reveals problems with cognitive representation of symptom identity, emotional representations of embarrassment and shame, and a subjective norm that women believe their symptoms will be trivialised by clinicians. Together these barriers frustrate women’s identification of their candidacy for healthcare. Addressing these issues through behavioural change interventions for women and clinicians, will help to achieve universal access to pelvic healthcare services (United Nations Sustainable Development Goal 3.7).

**Systematic Review Registration:**

PROSPERO CRD42021256956.

**Supplementary Information:**

The online version contains supplementary material available at 10.1186/s12905-024-03063-6.

## Background

Women’s health is finally emerging as a globally important subject. United Nations (UN) Sustainable Development Goals (SDG) 3.7 states we should “by 2030 ensure universal access to sexual and reproductive health care services, including for family planning, information and education, and the integration of reproductive health into national strategies and programmes” [1]. In the United Kingdom (UK), there is growing emphasis on promoting education on women’s health issues, reducing associated stigma, and increasing access to reliable information about women’s health [2, 3].

Many women’s health symptoms are considered difficult to talk about, both by women, health care professionals (clinicians) and the public in general [4, 5]. Stigma surrounding pelvic symptoms (including urogynaecological and bowel symptoms) matters because it stops women from seeking help. Symptoms such as urinary incontinence (UI) and prolapse can be addressed through early detection and timely receipt of conservative therapies such as pelvic floor muscle training [6, 7]. Although not life threatening, these pelvic symptoms are common: pelvic floor dysfunction (PFD) including urinary and faecal incontinence, bladder, bowel, and sexual dysfunction, prolapse and persistent pelvic pain, is prevalent in up to 50% of women [8], and has a significant impact on women’s quality of life and physical, mental, and social wellbeing [9, 10]. The intimate, personal and varied nature of pelvic symptoms, causes significant embarrassment and shame, leading to further psychological distress, reduced functioning, poor body image and social and occupational difficulties [9, 11–14].

Despite the widespread experience of pelvic symptoms, the number of women who seek healthcare is relatively low, as evidenced by most prevalence data on healthcare seeking related to UI. In a large population from the Nurses’ Health Study I and II, of 94,692 middle aged and older women with UI, only 34% reported discussing their symptoms with a clinician [15]. Similarly, in a web-based survey of 5,861 Danish women experiencing UI, only 29% had sought professional help [16]. In the UK, a postal evaluation of 2,414 women registered to a general practice found UI prevalent in 40% but only 17% sought professional help [14]. More stigmatized pelvic symptoms were included in an online survey of 376 Australian women: 99% had bladder, bowel, sexual dysfunction or prolapse, with 51% seeking help [5], but in the United States (US), only 29% of 938 women aged 45years or more with accidental bowel leakage sought care [17]. Two recent systematic literature reviews exploring experiences of prolapse, found that despite the availability of effective early treatment options, women lack knowledge and awareness about symptoms and available treatments [10, 18].

It is important to understand the barriers and facilitators women experience when seeking healthcare for stigmatized pelvic symptoms to develop approaches to increase knowledge and awareness among the public and clinicians, to encourage women to seek healthcare when necessary and design or redesign services to meet women’s needs. Literature on barriers and facilitators to help seeking with pelvic symptoms does exist but is spread across different conditions or symptom groups, settings, and populations, and has been generated using different methodologies. To our knowledge, this literature on barriers and facilitators has not been brought together systematically to share learning across different conditions, populations, and methodologies.

This systematic review aimed to identify the barriers and facilitators women in high income countries face in seeking help for stigmatised pelvic symptoms. We used the Common-Sense Model of Self-Regulation of Illness and Behaviour (CSM) [19–21], a model from health psychology, to synthesise and interpret the review’s findings as it helps explain how people behave (e.g. whether to seek help or not) in reponse to potential health threats (e.g. experience of symptoms or receipt of a diagnosis). The model argues that, on being faced with a possible health threat (such as pelvic symptoms), people are triggered to respond, which takes place in three stages. In Stage 1, people interpret or make sense of the threat in relation to previous experiences and their sociocultural environment, to form beliefs about what condition they have, its likely cause, consequences, duration, and cure/controllability (‘interpretation’). These beliefs are also accompanied by emotional responses to the health threat. In Stage 2, they decide how to cope with the threat (‘coping’), which may include going to a doctor, taking medication, self-care (‘approach coping’) or denial, wishful thinking (‘avoidance coping’). In Stage 3 they assess if their way of coping was effective in returning to a normal state of self (‘appraisal’). The model was recently extended to include people’s beliefs about the behaviour and treatment as determinants of coping procedures and illness outcomes, in addition to illness representations [22].

## Methods

The review is reported according to the Preferred Reporting Items for Systematic Reviews and Meta-Analyses Protocols (PRISMA) statement [23]. PRISMA checklists are available (Additional File 4). PROSPERO protocol registration number CRD42021256956.

The SPIDER (Sample, Phenomenon of Interest, Design, Evaluation, Research type) search structure [24] was chosen as the conceptual framework to specify the review question, develop selection criteria and design search strategy. Although its authors [24] found that SPIDER was not as sensitive as a traditional PICO [25], it has been recommended as a systematic and rigorous tool in reviews addressing non-quantitative research questions and offers an optimal balance between sensitivity and specificity in searching [26] and more easily managed results [24]. Table 1 shows the framework concepts.

**Table 1:** *SPIDER framework concepts*

S	SAMPLE	women from high income countries, with stigmatised pelvic symptoms
P I	PHENOMENON OF INTEREST	help, health, care, consultation, treatment or information seeking behaviour, intention, or perception; accessing healthcare, Women’s health services
D	DESIGN	any research design that uses data collection methods to capture participants’ help-seeking experiences (including, but not limited to, focus groups, interviews, questionnaires and surveys)
**E**	EVALUATION	experiences, attitudes, perceptions, life change events, emotions, views, knowledge, barriers, and facilitators
**R**	RESEARCH TYPE	qualitative, quantitative, and mixed methods

### Eligibility criteria

Included pelvic symptoms (Sample) were limited to those likely to affect quality, rather than length, of life. From the literature, pelvic symptoms associated with a degree of stigma in disclosure, and eligible for inclusion in this literature review, were prolapse [27], urinary and faecal incontinence [12, 28], sexual dysfunction, PFD [5], genital infections such as warts and herpes [29, 30], pelvic pain, and abnormal uterine bleeding [4, 31]. Some pelvic symptoms arise from issues such as intimate partner violence (IPV), rape, abortion, infertility, female genital mutilation, Human Immunodeficiency Virus/ Acquired Immunodeficiency Syndrome, Human Papilloma Virus, and urogynaecological cancers. These issues were excluded in favour of including the symptoms that may result from them. Table 2 shows the full list.

**Table 2:** *Included stigmatised pelvic symptoms*

Pelvic organ prolapse	Dyspareunia	Vaginal bulge	Genital herpes
Urinary incontinence	Genital symptoms	Perineal pain	Genital sores
Faecal incontinence	Urogenital symptoms	Perineal discomfort	Genital ulcers
Menopause	Urogynaecology symptoms	Vaginal discharge	Genital blisters
Sexual dysfunction	Urinary symptoms	Vaginal itch	Flatulence
Reproductive tract infections	Anal symptoms	Vulval itch	Constipation
Pelvic floor disorders	Vulval symptoms	Vaginal odour	Vaginal infection
Abnormal uterine bleeding	Vaginal symptoms	Anal discharge	Vulval infection
Pelvic pain	Vaginal pressure	Genital warts	Genital infection
	Perineal pressure		Urine infection

The Phenomenon of Interest was help-seeking and its alternative terms. Any study design that captured help-seeking views were included. Evaluation included barriers and facilitators that women expressed about seeking help. The ‘research type’ included peer reviewed, published, qualitative, quantitative, or mixed methods primary studies, set in high-income countries only. A summary of eligibility criteria is in Table 3.

**Table 3:** *Summary of eligibility criteria*

Inclusion Criteria	Exclusion Criteria
SAMPLE Studies of stigmatised pelvic symptoms among women from high income countries	Studies including males, unless female data can be separated Studies including other symptoms, conditions or issues, unless data for stigmatised pelvic symptoms can be separated IPV or rape; abortion; FGM; infertility; contraception HIV/AIDS or HPV or attending for HIV/HPV screening Cancer or attending for cancer screening Studies that focus on clinicians, health service managers’, or carers’ views
PHENOMENON OF INTEREST Studies exploring help seeking	Studies that do not explore help seeking Studies that include seeking help other than for pelvic symptoms, unless these can be separated Studies that focus on seeking help other than for health reasons (e.g., justice) Studies that focus on treatment decision making after seeking help
DESIGN Studies of any design using data collection methods to capture participants’ help-seeking views or experiences (including but not limited to focus groups, interviews, questionnaires, and surveys), and including those reported in systematic reviews	
EVALUATION Studies incorporating participants’ emotions, attitudes, perceptions, barriers, issues, problems, difficulties, facilitators, enablers, life change events, beliefs, feelings, knowledge, and understanding in relation to seeking help	Studies reporting only prevalence rates or predictors for help seeking
RESEARCH TYPE Peer reviewed qualitative, quantitative, or mixed methods primary studies	Unpublished, non-peer reviewed, ‘grey literature’, conference abstracts
OTHER *Context*: include studies set in countries with economies similarly developed to the UK (World Bank 2021 Country Classification “high income”) *Language*: include studies written in English, German, French, Spanish, and Swedish *Date of publication*: from the year of inception of the database searched, to the date of the search	Studies set in any other countries Studies written in any other language

### Information sources

Databases were searched using the platform EBSCOhost: MEDLINE, CINAHL complete, PsycINFO, SocINDEX with Full Text; PubMed, and the Cochrane Database of Systematic Reviews (CDSR), Cochrane Central Register of Controlled Trials (CENTRAL); primary studies included in topic relevant systematic reviews; reference list checking of included studies; forward citation searching of included studies in Scopus. Studies were included from year of inception of databases searched, to May 2023.

### Search strategy

Scoping searches, MeSH headings used in known relevant studies, thesaurus, and the author’s clinical experience were used to identify subject headings and key words for pelvic symptoms, barriers, and facilitators to seeking healthcare. Peer Review of Electronic Search Strategies (PRESS) checklist [32] was applied by a medical information specialist. Ethical approval was not sought because this review synthesised results from primary research studies already published. The final search included a combination of terms related to two main concepts: stigmatised pelvic symptoms (Sample) AND help seeking (Phenomenon of Interest). Table 4 shows an example of the search strategy used in MEDLINE. The search strategy was translated by hand for the other databases and registers searched.

**Table 4:** *An Example of the Search Strategy in MEDLINE*

**SAMPLE** MeSH (MM “Pelvic Organ Prolapse+”) OR (MM “Urinary Incontinence+”) OR (MM “Fecal Incontinence”) OR (MM “Vaginal Discharge+”) OR (MM “Menopause+”) OR (MM “Sexual Dysfunction, Physiological+”) OR (MM “Sexual Dysfunction, Psychological+”) OR (MM “Reproductive Tract Infections”) OR (MM “Pelvic Floor Disorders”) OR (MM “Pelvic Pain+”) OR (MM “Dyspareunia”) OR (MM “Condylomata Acuminata”) OR (MM “Herpes Genitalis”) OR (MM “Flatulence”) OR (MM “Constipation+”) OR **Keywords** TI pelvic organ prolapse OR AB pelvic organ prolapse OR TI prolapse OR AB prolapse OR TI urinary incontinence OR AB urinary incontinence OR TI f#ecal incontinence OR AB f#ecal incontinence OR TI incontinen* OR AB incontinen* OR TI Menopause OR AB Menopause OR TI sexual dysfunction OR AB sexual dysfunction OR TI reproductive tract infection* OR AB reproductive tract infection* OR TI Pelvic floor disorder* OR AB Pelvic floor disorder* OR TI Abnormal uterine bleeding OR AB Abnormal uterine bleeding OR TI Pelvic pain OR AB Pelvic pain OR TI Dyspareunia OR AB Dyspareunia OR TI genital symptom* OR AB genital symptom* OR TI urogenital symptom* OR AB urogenital symptom* OR TI urogyn#ecolog* symptom* OR AB urogyn#ecolog* symptom* OR TI urinary symptom* OR AB urinary symptom* OR TI anal symptom* OR AB anal symptom* OR TI vulva* symptom* OR AB vulva* symptom* OR TI vagina* symptom* OR AB vagina* symptom* OR TI vagina* pressure OR AB vagina* pressure OR TI pressure N3 vagina* OR AB pressure N3 vagina* OR TI pressure N3 perine* OR AB pressure N3 perine* OR AB vagina* bulge OR TI perine* pain OR AB perine* pain OR AB perine* discomfort OR TI vagina* discharge OR AB vagina* discharge OR AB vaginal itch OR AB itch N3 vagina* OR AB itch N3 vulva* OR AB vaginal odo#r OR AB anal discharge OR TI genital wart* OR AB genital wart* OR TI genital herpes OR AB genital herpes OR TI genital sore* OR AB genital sore* OR TI genital ulcer* OR AB genital ulcer* OR TI genital blister* OR AB genital blister* OR TI flatulen* OR AB flatulen* OR TI constipat* OR AB constipat* OR TI genital infect* OR AB genital infect* OR TI vaginal infect* OR AB vaginal infect* OR AB vulva* infect* OR TI urin* infect* OR AB urin* infect* AND **PHENOMENON OF INTEREST**
**MeSH** (MM “Help-Seeking Behavior”) OR (MM “Information Seeking Behavior”) OR (MM “Health Services Accessibility+”) OR (MM “Women’s Health Services+”) OR (MM “Patient Acceptance of Health Care”) OR **Keywords** TI ((help-seeking OR help seeking) behavio#r*) OR AB ((help-seeking OR help seeking) behavio#r*) OR TI ((care-seeking OR care seeking) behavio#r*) OR AB ((care-seeking OR care seeking) behavio#r*) OR TI ((treatment-seeking OR treatment seeking) behavio#r*) OR AB ((treatment-seeking OR treatment seeking) behavio#r*) OR TI ((health seeking OR health-seeking) behavio#r*) OR AB ((health seeking OR health-seeking) behavio#r*) OR TI ((help-seeking OR help seeking) intention*) OR AB ((help-seeking OR help seeking) intention*) OR AB ((care-seeking OR care seeking) intention*) OR TI ((information-seeking OR information seeking) behavio#r*) OR AB ((information-seeking OR information seeking) behavio#r*) OR TI ((information-seeking OR information seeking) intention*) OR AB ((information-seeking OR information seeking) intention*) OR TI ((knowledge-seeking OR knowledge seeking) behavio#r*) OR AB ((knowledge-seeking OR knowledge seeking) behavio#r*) OR TI ((knowledge-seeking OR knowledge seeking) intention*) OR AB ((knowledge-seeking OR knowledge seeking) intention*) OR TI (perception of (help seeking OR help-seeking)) OR AB (perception of (help seeking OR help-seeking)) OR TI (perception of (care seeking OR care-seeking)) OR AB (perception of (care seeking OR care-seeking)) OR TI (perception of (treatment seeking OR treatment-seeking)) OR AB (perception of (treatment seeking OR treatment-seeking)) OR AB (perception of (information seeking OR information-seeking)) OR TI seek* consultation OR AB seek* consultation OR TI ((health service* OR healthcare OR health care) access*) OR AB ((health service* OR healthcare OR health care) access*) OR TI Women* Health Service* OR AB Women* Health Service*

### Selection process

After removing duplicates, all retrieved studies were screened by title and abstract by two independent reviewers. 10% of full texts were independently dual screened, with substantial agreement (83%; prevalence and bias adjusted kappa [PABAK] 0.66). Study authors were contacted by email where information was unclear or appeared missing, with a response time of three weeks, after which studies were excluded.

### Data extraction

A data extraction form designed using Excel, with data items informed by Noyes, Booth [33] and NICE [34]. was reviewed and piloted by the research team. Data were extracted by the author, and independently from 33% of included papers by a research assistant. Quantitative data on barriers and facilitators to help-seeking were copied verbatim into the data extraction form and narratively summarised. Qualitative data recording participants’ help seeking views or experiences, found in results or discussion sections, were copied verbatim into NVivo software for analysis.

### Quality assessment

The Mixed Methods Appraisal Tool (MMAT) [35] was used to appraise the methodological quality of each study by the author, and jointly for 33% of studies by a research assistant. The MMAT is pilot tested, interrater reliability tested, and offered five study design categories (one qualitative, three quantitative and one mixed methods) with five core criteria. Information about which areas of a study were problematic are reported, rather than summative scores because this gives more detail.

### Data analysis and synthesis

Quantitative data were narratively synthesised, with content analysis of barriers and facilitators, and discussed and agreed with co-authors. Primary qualitative data were extracted and imported into NVivo software, before coding into pre-existing concepts from the analysis of quantitative data, with new concepts added as necessary. Reflecting on patterns and meaning in the data, themes were generated, developed, and reviewed at length through reflective thematic analysis [36], sense-checked with co-authors, and refined before naming and definition. Quotations from participants were used to illustrate themes. Synthesis of quantitative, qualitative, and mixed methods results drew together themes about barriers and facilitators to healthcare seeking with stigmatised pelvic symptoms, which were mapped to the CSM. Mapping the data to theory helped to explain the relationship of identified themes to help-seeking behaviours and identify potential targets for intervention.

### Assessment of confidence in cumulative evidence

Grading of Recommendations Assessment, Development and Evaluation - Confidence in Evidence from Reviews of Qualitative research (GRADE-CERQual) [37], was used to assess confidence in the findings in terms of methodological limitations, relevance to the review aim, coherence of the review findings in relation to the primary data, and the adequacy of data presented in the primary studies. The Data Richness Scale [38] was used to assess adequacy of qualitative data.

**Reflexivity.** The authors have backgrounds in pelvic health physiotherapy, with lived experience (CJ), applied health research (PA, MM), health psychology (PA) and sociology (MM). Before conducting the review, the authors considered their own philosophical positions, context, and life experiences in discussion with each other, to facilitate transparency of relevant preconceptions and beliefs.

## Results

### Results of search

**Fig. 1:**
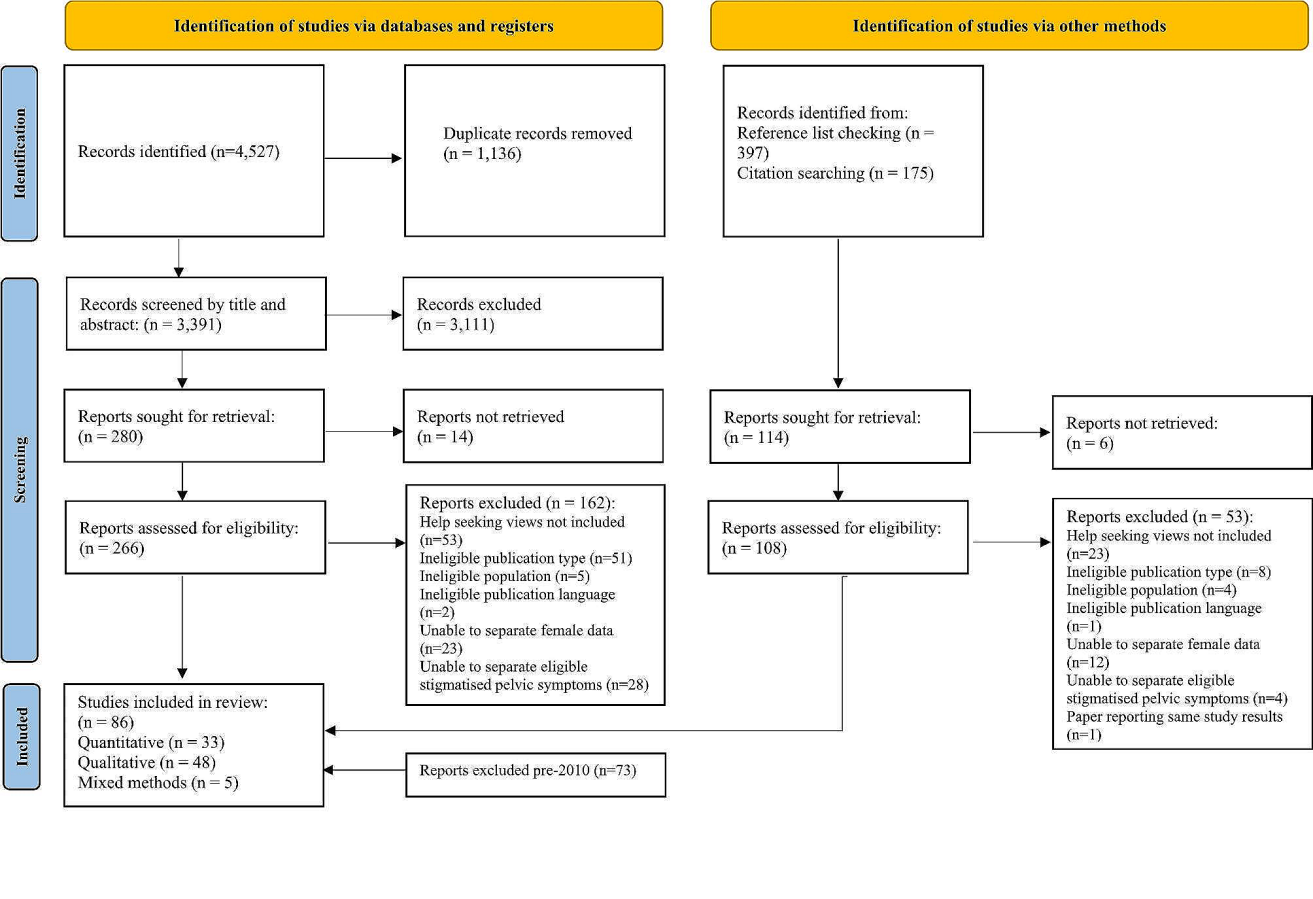
Prisma flow diagram

The electronic search generated 4,527 papers, and reference list checking and forward citation searching found 572 papers. After removal of duplicates, 3,963 titles and abstracts were screened, of which 3,569 were excluded, leaving 394 studies. It was not possible to access 20 papers, and eligibility criteria were not met by 215 papers after full text screening, leaving 159 papers that met all inclusion criteria (53 quantitative, 101 qualitative, 5 mixed methods). Initially, studies were not excluded based on publication year. However, it became apparent that the publication year of included studies ranged from 1988 to 2023, with 48.3% published between 1988 and 2010. This range encompassed a period of significant technological and cultural change, that occurred following the turn of the millennium (e.g., emergence of world wide web). It was speculated whether women’s experiences of barriers and facilitators were the same or had changed due to developments and cultural changes over this period. To test this speculation, data from all quantitative studies were extracted, and content analysis used to code healthcare seeking barriers and facilitators. These were compared across five decades from the 1980s to the present and were found to be similar. This suggested that excluding papers before 2010 was unlikely to miss barriers to healthcare seeking that are currently important to women. Exclusion of 73 studies prior to 2010 led to a total of 86 studies included in this review (33 quantitative, 48 qualitative, and 5 mixed methods). Figure 1. shows the search results displayed in a PRISMA flow diagram.

### Overview of studies

The main characteristics of quantitative, qualitative, and mixed methods studies are available (Additional File 1). Broadly, 36.05% of papers were from Europe, 31.40% from North America, 20.93% from East Asia and Pacific, 6.98% from Middle East and North Africa, 2.33% Worldwide, and 1.16% from Latin America. The geographical representation of all included studies is shown in Table 5. Participants in all studies were described as ‘women’ or ‘female’: whilst recognising that not everyone with female anatomy identifies as a woman, or female, we have used these terms throughout this paper.

**Table 5:** *Geographical Representation of Included Studies*

	Quantitative		Qualitative/Mixed		
Countries	No. of women represented	No. of papers	No. of women represented	No. of papers	References
UK + Ireland	593	2	852	8	[27, 39–47]
Norway, Denmark, Belgium, and Portugal	1,940	1	-	-	[48]
Denmark	4,051	1	14	1	[49, 50]
Norway	-	-	15	1	[51]
The Netherlands	822	2	62	4	[12, 52–56]
Spain	-	-	18	1	[57]
Italy	-	-	6	1	[58]
Sweden	561	1	77 + 16 blog posts	5	[59–64]
Germany and Denmark	1,849	1	-	-	[65]
Germany	-	-	71	2	[66, 67]
Poland	141	1	16	1	[68, 69]
United Arab Emirates	556	2	-	-	[70, 71]
Saudi Arabia	794	3	-	-	[72–74]
Israel	223	1	-	-	[75]
USA	3,877	11	1,055	16	[9, 31, 76–99]
Chile	-	-	10	1	[100]
Hong Kong	639	1	30	1	[101, 102]
Korea	500	1	-	-	[103]
Singapore	95	1	-	-	[104]
Japan	774	1	-	-	[105]
Macau SAR, China	408	1	-	-	[106]
Taiwan	-	-	38	2	[107, 108]
Australia	1,362	2	293	7	[5, 109–116]
New Zealand	-	-	10	1	[117]
Worldwide	-	-	70 + 200 blog posts*	2	[118, 119]

Note: *200 online posts were randomly chosen from 985 posted by 762 unique users on 98 websites [118]

Quantitative studies (*n* = 33) represented 19,185 female participants from nineteen high income countries. All but one study used cross sectional survey design with questionnaires, mostly using unvalidated, bespoke questions on healthcare seeking. Due to heterogeneity of methods, meta-analysis was not possible. One study used a discrete choice experiment to investigate if cost of care and appointment wait time affected healthcare seeking intentions with urinary tract infection (UTI) symptoms [76]. Stigmatised pelvic symptoms studied included urinary incontinence (18 papers), PFD (five papers), sexual dysfunction (three papers), prolapse (two papers), pelvic pain, urinary tract infection, uro-genital atrophy, constipation, and menstrual dysfunction (one paper each).

Qualitative (*n* = 48) and mixed methods (*n* = 5) studies represented views and experiences of 2,653 women collected through interviews, focus groups, and 216 blog posts, from fifteen high income countries. Only eight papers stated the theoretical framework on which their study was based. Stigmatised pelvic symptoms studied included urinary incontinence (19 papers), PFD (nine papers), prolapse, and pelvic pain (eight papers each), urinary dysfunction, and sexual dysfunction (six papers each), anal incontinence (two papers), and mixed urinary and anal incontinence (two papers).

### Quality Assessment

Using the MMAT indicated that 19 of 33 quantitative papers lacked information about representativeness of the sample. Authors were contacted for clarification, with few responses. Ten quantitative papers lacked appropriateness of measures for the healthcare seeking element, possibly because healthcare seeking was often a secondary theme. 28 quantitative papers did not provide any, or enough information on reasons for non-participation, resulting in an uncertain risk of non-response bias. All MMAT criteria were met in 37 of the 48 qualitative papers. In five papers it could not be established if the findings were adequately derived from the data, and in nine papers there was not enough information to determine coherence between data sources, collection, analysis, and interpretation. The interpretation of results was not substantially derived from data in five papers. Data Richness Scale assessments showed 40 qualitative papers had reasonable to good amounts and depth of data. No papers were excluded based on their data richness score. Each theme was assessed for data ‘adequacy’. Most had only minor concerns meaning that there were many studies within a theme, some with only little or superficial data, but some more detailed and specific. Mixed methods papers met all the qualitative methodological quality criteria but there were limitations in quantitative methodological quality in all five studies, and in mixed methods methodological quality in all but one paper. Most frequently this was uncertainty about different components of the study adhering to the quality criteria of each tradition of the methods involved. Quality assessment of all studies using the MMAT is accessible (Additional File 2). The CERQual assessment of confidence in the evidence across the key themes was high, with no, or minor concerns about methodological limitations, coherence, relevance, and data adequacy. The results of quality assessment suggest the need for higher quality research in quantitative descriptive studies in this field, particularly to facilitate the assessment of risk of nonresponse bias.

### Outcomes

#### Quantitative studies

The most cited barriers were coded as embarrassment, shame, and taboo, (18 papers) closely followed by participants expressing a lack of knowledge about where to seek healthcare, and about treatment options, with a low expectation of benefit (18 papers). Some participants indicated that they did not recognise their symptoms as a significant medical problem, or thought their symptoms were not troublesome enough to seek healthcare and deprioritised them (19 papers). Many thought their symptoms were normal, especially after childbirth, or with ageing (15 papers). Participants frequently reported that if their clinician asked at all, they were embarrassed, were not interested in, or would not take their pelvic symptoms seriously (14 papers). Others perceived their clinician was too busy and did not want to bother them about pelvic symptoms (5 papers). Fear of being examined, and of required investigations and treatment, were barriers (17 papers), with a few participants being fearful that their symptoms indicated more serious disease (3 papers). Waiting times, inconvenience, being too busy to attend, transport issues, religious, and cultural factors, language difficulties and service issues such as appointment delays, and cost, were all obstacles (21 papers). A less common barrier to seeking healthcare was a desire to cope or self-help (5 papers).

Facilitators for seeking healthcare most often included increased bother from pelvic symptoms (9 papers). Support from family and friends to seek healthcare (4 papers), and knowledge and learning about new treatments encouraged some participants (3 papers), whilst others only sought help due to stigma, embarrassment, self-blame, guilt, or depression about their pelvic symptoms (3 papers) or feared that their symptoms were indicative of serious disease (2 papers). Papers containing the key barriers and facilitators are referenced (Additional File 3).

#### Qualitative and mixed methods studies

Four themes encompassed women’s barriers to healthcare-seeking: (1) Stigma, (2) Women’s lack of knowledge (with three sub-themes of normalising, deprioritising, and fear), (3) Trivialising by clinicians, and (4) Inconvenience and cost of seeking healthcare.

**Stigma** this theme was a key barrier to help-seeking, encapsulating the frequently used codes, “embarrassing’, “ashamed’, and less often, ‘taboo” (30 papers).

*“For me, I was embarrassed to speak to anybody, really, about it, for a long time. But now, I regret that I did that, because I left myself to a bad stage.”* [prolapse]; [27]

*“You don’t know why, you feel sort of ashamed, you feel embarrassed to talk about it, as if you are somehow a failure, with guilt, you know?”* [47 years with UI]; [57]

*“Yes. You can talk about almost anything else I think, all kinds of matters considering your genitals and. but not this, this I think is very taboo”* [SUI] [59].

Embarrassment is the emotional impact from stigma, with shame also associated with stigma [120]. Stigma may be categorised as enacted or felt. Felt stigma may be internalised, perceived, and anticipated [121]. Internalised stigma was most often described by women seeking help with stigmatised pelvic symptoms, in the way they internalised negative beliefs and perceptions around their symptoms, expressed psychological distress, reduced self-worth, shame, and self-loathing [122]. Some participants expressed greater embarrassment to talk to a male clinician: “*…My GP is a handsome 40-year-old man, and I would not dream of [laughs] talking to him about anything like that!”* [sexual dysfunction] [51], while others blamed themselves for their symptoms: “*When I was younger, I took a lot of laxatives, so I did this to myself*” [bowel leakage] [78], or felt self-disgust: “…*I feel dirty and disgusted in myself already*” [bowel leakage] [115].

**Lack of knowledge** about symptoms in general caused many participants uncertainty over whether to seek healthcare (23 papers):

*“You feel disoriented, you don’t know if it is normal or not, whether you should worry or not”* [45 years with UI] [57].

*“I did not know that happened to women. I did not know anything about it. I was scared because I didn’t know what it was.”* [prolapse] [98].

“*How can you talk about something [when] you don’t even know what it is?”* [bowel leakage] [78].

Three sub-themes related to ‘lack of knowledge’: normalising, deprioritising, and fear. ‘***Normalising***’: participants normalised pelvic symptoms as women, following childbirth, and with ageing, as something they should not seek medical help for (22 papers):

*“I simply thought: the urinary incontinence is just part of it. Your whole body is turned inside out after delivery anyway. So I thought it’s just part of the game.’*” [PFD] [12].

*“I have some good friends, and my daughter. Well, they have the same problem. It’s age. That’s all we boil it down to is the age. Nothing you can do about it.”* [urinary dysfunction] [85].

‘***Deprioritising***’ was developed from new codes in qualitative data relating to prioritising other things, avoiding, or denying pelvic symptoms, and low bother from symptoms, which was found across all data (19 papers):

“*We forget about ourselves a little. Everybody else comes first, and then later, me.”* [PFD] [84]. Participants across a wide range of pelvic symptoms felt low symptom bother did not justify seeking help: “…*it’s only a little bit, not like oh I’ve wet my pants”* [urinary dysfunction] [47] and “*I just forget about it, because it’s not an every week thing.”* [bowel leakage] [78].

‘***Fear***’ related to women’s lack of knowledge and information and included codes about fear of examinations, investigations, and treatments, and inappropriate fear of serious disease, all of which delayed seeking help (8 papers):

*“To be exposed, that is something you don’t want to risk, so every time [examination] it is like a mental procedure, the sense of exposure. Well, it’s almost like an abuse, it is something you don’t want to do but you must.”* [pelvic pain -endometriosis] [61].

“*I didn’t want to be put on some pill that would make me more constipated. Sometimes the cure is worse than the disease…”* [bowel leakage] [78].

“*When your uterus or bladder falls, it is very dangerous. You can get cancer*” [PFD] [86].

‘**Trivialising**’ was a significant theme that grew around codes involving women’s relationship difficulties with their clinician (25 papers). A new code from qualitative data included in this theme was women feeling judged by clinicians if they mentioned pelvic symptoms. Women felt they were not being taken seriously, not being asked about symptoms, and perceived their clinician was embarrassed to discuss symptoms:

“*I told my doctor, I had urine loss all the time…you know what he said? Honestly, I will tell you…”wear a kotex””* [PFD] [86].

“*You’ve got a rectocele.’ ‘What is it?’ ‘Oh, you don’t need to know.’ Well, hey, if it’s to do with you, you’re the one person who needs to know about it. You shouldn’t be sort of kept like, ‘Oh, you’re a child being a nuisance. Go away. You don’t need to know.”* [prolapse] [87].

*“And then she also said that maybe I should learn to live with it, I thought that was a bit crazy. And ehm, that also made me think I did not feel taken seriously. Because I really thought, well, hello, I’m 20!”* (22 yrs) [pelvic pain – vulvodynia] [52].

*“The lack of urgency is real with OBGYNs. Maybe younger doctors are more open, but the attitude of older gynaecologists is to do what they did to me. He just gave me a pat on the butt and told me I could live with it.”* [prolapse] [90].

‘Trivialising’ also included women expressing their perception that their clinicians lacked knowledge or training about pelvic symptoms, found in two quantitative and 12 qualitative and mixed methods studies:

“*The GP took me seriously, but in retrospect I think he didn’t have the knowledge…”* [pelvic pain – vulvodynia] [52].

**‘Inconvenience and cost of seeking healthcare**’ developed as a theme from overlapping codes in which women described a variety of cultural, gender or religious factors, as well as communication issues with their clinician, long waiting times at appointments making them difficult to fit in to everyday life, and for some, the cost of having to take time away from paid work, or childcare to attend, as barriers to help-seeking (15 papers). Codes around service issues were incorporated: the inconvenience women experienced to physically attend appointments or have treatment, delays in receiving an appointment for a particular service, and the cost of care, especially if they did not have health insurance (12 papers).

Facilitator codes only found in qualitative and mixed methods studies included clinicians taking women seriously, being open to uncertainty, asking about symptoms, and offering support, developing the new theme of ‘supportive clinician attitude’(18 papers), which was added to the themes of worsening symptoms, increasing women’s knowledge, and social support already found in quantitative studies.

#### Synthesis of all results

Table 6 shows how themes were developed from codes across the data. There was high certainty from the data that barriers and facilitators to healthcare seeking were similar across different stigmatised pelvic symptoms, countries, and research designs.

**Table 6:** *Codes and themes developed from all data on barriers to seeking healthcare with stigmatised pelvic symptoms*

Code	Theme/ sub-theme	Overarching Theme
Embarrassment about symptoms Embarrassment to talk to a [male] clinician Shame about symptoms Feeling symptoms are taboo Self-blame, self-disgust, self-stigma	Stigma	UNCLEAR CANDIDACY FOR HELP SEEKING [123, 124]
Unaware that treatments are available Not knowing where to seek help Low expectations of treatment benefit Hoping symptoms go away on their own	Women’s lack of knowledge about pelvic symptoms: - *General*	
Thought symptoms were normal Thought symptoms were normal after childbirth Thought symptoms were normal for ageing	- *Normalising*	
Prioritising other things Denial/ avoidance of symptoms Did not feel symptoms were a medical problem Did not feel symptoms were troublesome enough Inappropriate self-help/ coping	- *Deprioritising*	
Fear of clinician, examination, investigation, medication, surgery Fear of discovering a serious disease	- *Fear*	
Can’t afford cost of healthcare/ no insurance Women too busy to attend appointments Waiting time too long Language issues Transport issue to get to appointment	Inconvenience and cost of seeking help	
Feeling clinician was not interested/ would not pay attention Feeling clinician did not take symptoms seriously Clinician did not ask about pelvic symptoms Perceived that clinician was embarrassed to ask Felt judged by clinician Women believe clinicians lack training/knowledge Clinician did not give information about diagnosis	Clinicians trivialising symptoms	

The extended CSM was applied to better explain these results by describing how women’s perceptions about, and interpretation of their symptoms influence their behaviours in relation to coping with those symptoms. Women’s interpretation of symptoms is influenced by the cognitive and emotional representations triggered by their symptoms, which may be influenced by previous experiences, and sociocultural factors. Accurate cognitive representation of the potential threat from pelvic symptoms requires women to know the identity, cause, consequences, cure/controllability, and likely timeline of their symptoms. Findings from this review suggest that women’s lack of knowledge, reported in 44 studies, and normalising of symptoms, reported in 37 studies, threaten identification of pelvic symptoms. Attribution of cause is threatened by women believing their symptoms are normal. In the early stages, the full consequences of pelvic symptoms may not be appreciated because initially symptoms cause low bother and are deprioritised and normalised. Conversely, some women delay healthcare seeking because they (usually incorrectly) fear serious disease because of their symptoms. Lack of knowledge of treatment options threatens appropriate representation of the timeline and cure/controllability of pelvic symptoms, with some women hoping for spontaneous resolution, whilst others believe their symptoms are incurable. Women’s ability to make sense of their perceptions (coherence) of symptoms is affected by a lack of knowledge, that disrupts women’s cognitive representation of their symptoms.

Women’s main emotional representation of the potential threat from pelvic symptoms is stigma (embarrassment, shame, and taboo). Cited in 52 studies, stigma was the most reported barrier to healthcare seeking, and to a lesser extent, fear: of examination, investigations, treatment, and serious disease.

**Fig. 2:**
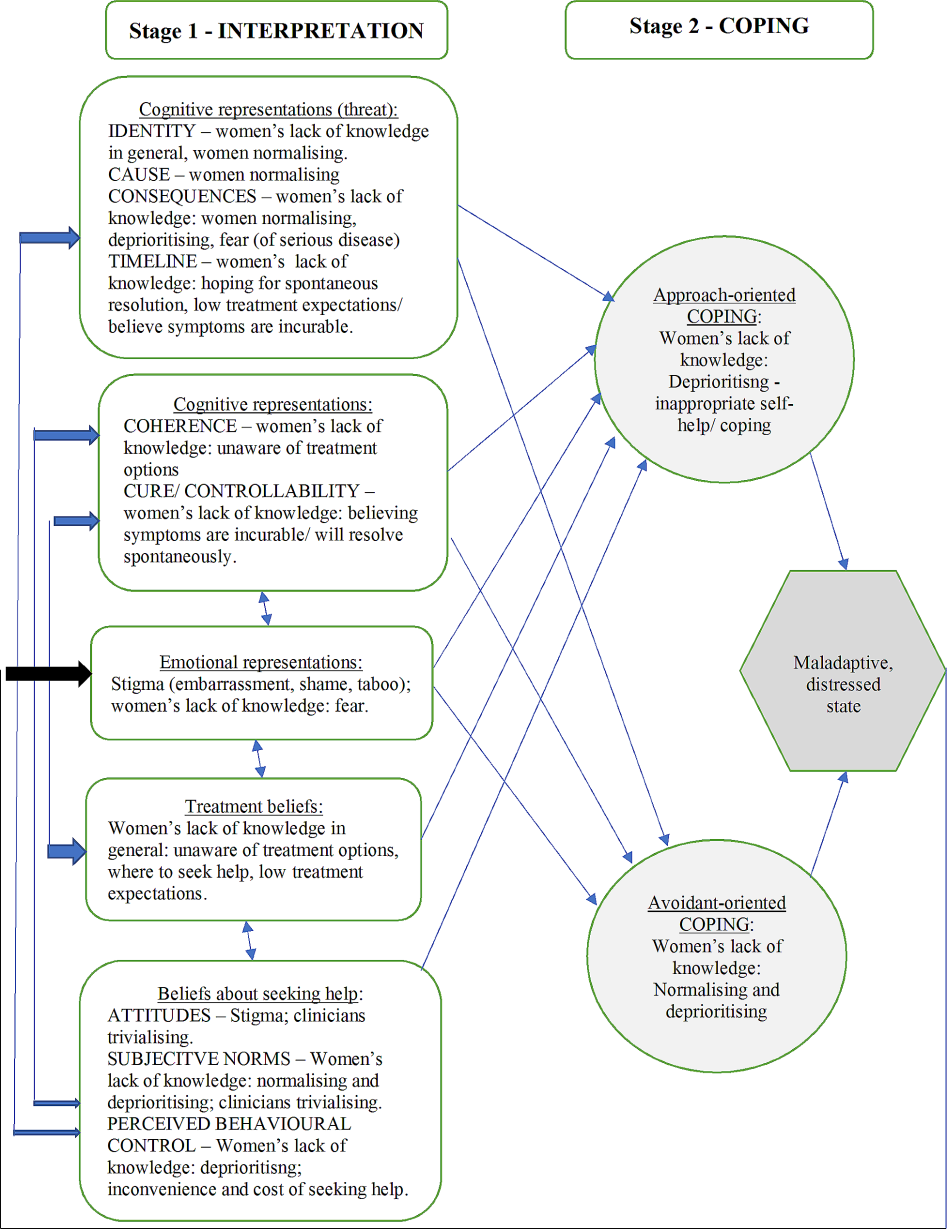
Using the extended CSM to explain barriers to healthcare seeking with stigmatised pelvic symptoms

Women’s treatment beliefs are affected by a lack of knowledge about treatment options, where to seek healthcare, and low expectations of treatment benefit, all delaying healthcare seeking. Women’s beliefs about seeking healthcare are influenced by sociocultural factors (subjective norms, perceived behavioural control), their own, and others’ attitudes. Attitudes of women seen in the data from this literature review indicated that women believed that seeking help for pelvic symptoms would cause them to feel stigmatised, that they would be judged, and their symptoms trivialised by their clinician, and that clinicians would normalise their symptoms, possibly due to a perception that clinicians lacked knowledge and training about pelvic symptoms. Subjective norms define what women believe others would do if they had pelvic symptoms: our data suggest the subjective norms are to normalise and deprioritise their own symptoms, cope, and feel stigmatised. Women’s perceived behavioural control over pelvic symptoms is reduced by lack of knowledge, service issues, and is affected by inappropriate self-help and coping. The key themes from help seeking barriers mapped to the CSM are shown in Fig. 2.

In summary, women’s cognitive and emotional representations, treatment beliefs, and beliefs about help-seeking affect their ability to manage their pelvic symptoms. The data show how coping (Stage 2. CSM) is affected by women’s lack of knowledge, causing (mis-)interpretation of their symptoms, and leading them to display either ‘approach-oriented coping’ through inappropriate self-help, such as relying on sanitary pads for incontinence, or ‘avoidant-oriented coping’ procedures such as normalising, and deprioritising symptoms, instead of seeking help. Women appear to become stuck in a maladaptive, distressed loop between the interpretation and coping stages of the CSM, because iterative interpretation of their symptom perceptions, and the social messages they gather about seeking help with pelvic symptoms, reinforce the stigma of, and their lack of knowledge about symptoms. For many women, it was only worsening impact from symptoms and fear of more serious disease that pushed them to seek healthcare. There were a small number of voices (six papers), who believed they should assert themselves to take responsibility to ask for professional help, but the majority of women suggested that a supportive attitude from their clinician, especially to ask women about pelvic symptoms, would facilitate seeking healthcare for stigmatised pelvic symptoms.

## Discussion

This is the first review which covers such a wide range of stigmatised pelvic symptoms, to our knowledge. The principal findings of this mixed methods systematic literature review are that stigma (embarrassment, shame, and taboo), lack of knowledge, and women feeling ‘trivialised’ by clinicians, are definitive barriers to seeking help. Using a health psychology model (CSM) contributed to understanding how the emotional representations (stigma) and cognitive representations (lack of knowledge) particularly affect identification of pelvic symptoms, and clinician behaviour. Referring again to theory, Dixon-Woods, Cavers [123] described the construct of Candidacy, to explain how, influenced by their context, other people, and sociocultural issues, individuals negotiate their eligibility for healthcare between themselves and healthcare services, in an iterative cycle. When someone seeks healthcare, they assert their candidacy, which is then judged by clinicians (‘Adjudication’), either helping, or hindering their healthcare journey. In the case of seeking help with pelvic symptoms, stigma, women’s lack of knowledge, and their experiences leading to an expectation of their symptoms being trivialised, combine to make women’s candidacy for healthcare unclear. If clinicians lack knowledge and training about pelvic symptoms, they may trivialise, normalise, or judge symptoms, and so adjudicate against women’s healthcare seeking attempts.

‘Unclear candidacy’ is proposed as the overarching theme for this synthesis. The connection between the Candidacy model and the CSM’s illness representations was demonstrated in a paper exploring access to, and experiences of healthcare services [124]. This connection helps to understand the voices of women seeking help in this analysis: Stigma, lack of knowledge, and feeling trivialised by clinicians were the key factors affecting women’s identification of themselves as candidates for healthcare. Women both judge their own symptoms and feel judged by clinicians as unsuitable, or unworthy, to seek help for stigmatised pelvic symptoms. Women’s beliefs that if they seek healthcare they will not be taken seriously by clinicians, collude to frustrate their candidacy for healthcare. Our data show that women experience felt stigma, and enacted stigma from negative judgements by clinicians, further discriminating against women’s candidacy for healthcare with stigmatised pelvic symptoms. The facilitators that most often prompted women to seek healthcare were more knowledge about pelvic symptoms, worsening symptoms, and feeling that their clinician was supportive, especially in asking specifically about pelvic symptoms. This suggests that women who believe their clinician will have a supportive attitude are more likely to develop a positive emotional representation of their symptoms and will more likely seek healthcare. Increasing women’s knowledge would help them to appropriately identify the cognitive representation of threat posed by their symptoms, to decide if they can appropriately self-manage their symptoms or need to seek professional help.

The strengths of our review are the inclusion of a wide range of carefully considered, stigmatised pelvic symptoms, explored across many high-income countries, with rigorous application of eligibility criteria, and the use of theoretical models to explain the link between barriers and facilitators and help-seeking behaviours, allowing suggestion of possible targets for intervention. Selection bias was reduced by the ability to include studies published in English, German, French, Spanish, and Swedish. Ethnic representation where reported, was mostly white and also included Black, Hispanic and Asian women. The overall CERQual assessments of confidence [37] were high for the barriers to healthcare seeking found in our review, signifying issues common to women across stigmatised pelvic symptoms. Help-seeking barriers concur with those found in recent systematic literature reviews investigating experiences of individual, stigmatised pelvic symptoms: abnormal uterine bleeding [4], prolapse [10, 125], and a recently published study exploring women’s experiences of PFD [93, 126], and urogynaecological care for racial and ethnic minority women [127]. Stigma, and lack of knowledge were likewise barriers for those with urinary incontinence [128, 129]. In a public survey, which was part of a call for evidence to inform the Women’s Health Strategy for England [130], published after commencement of this review, 84% of respondents said they had not been listened to by healthcare clinicians, which concurs with our findings, although not specific to pelvic health. Our finding that women perceived clinicians lacked knowledge and training (cited in 12 qualitative and mixed methods studies) was only found in one recent review relating to prolapse [10]. Our finding of women’s perception that clinicians normalise their pelvic symptoms (cited in seven qualitative and mixed methods studies), was only found in one review about abnormal uterine bleeding [4]. Few facilitators to healthcare seeking were reported in other reviews. Increased knowledge, social support and worsening symptoms were similarly found to encourage women to seek healthcare with PFD [10, 127, 131]. In contrast to others’ results, we found a large volume of qualitative data expressing the importance of a supportive clinician to facilitate women’s healthcare-seeking for pelvic symptoms. This may be due to the large number of women’s voices represented over a wide range of pelvic symptoms. It is likely to be an important consideration in developing future interventions.

We recognise limitations in this review. Although our search included many stigmatised pelvic symptoms, some relevant publications may have been missed, and not all symptoms were represented in the included literature. Grey literature was not investigated because we chose to include only peer reviewed studies to ensure a degree of rigour, and due to resource restrictions. Only women living in high income countries were included, to allow better understanding of barriers and facilitators in countries with similar economies to the UK, whilst recognising that the UK National Health Service is unique. Excluding studies published before 2010 is mitigated by thorough content analysis of the data in all quantitative studies concerning barriers and facilitators prior to exclusion, confirming that issues that currently concern women were unlikely to be missed. Quality appraisal using the MMAT was challenging because non-response bias was unclear in many quantitative studies, there was insufficient focus on healthcare seeking in ten papers, and few contacted authors responded to requests for clarification. Most included studies only captured the voices of women already seeking healthcare with symptoms: taking a public health approach to seek the concerns of all women may uncover further barriers and facilitators to seeking help for stigmatised pelvic symptoms not found in this review.

## Conclusions

The findings of this review mean that efforts to encourage women to seek healthcare with pelvic symptoms need to target the barriers by reducing stigma, increasing knowledge, and supporting primary care clinicians to routinely discuss stigmatised pelvic symptoms with women. Changing the social norm so women believe they will be taken seriously if they seek healthcare is likely to empower them to appropriately manage their symptoms. Since this review began, there has been an explosion of interest and information about menopause, with celebrity endorsement in the UK [132], which along with the first ever UK Government Women’s Health Strategy [130], may help to normalise discussion of stigmatised pelvic health symptoms, reducing stigma. Clinicians at all levels, particularly in primary care, need to legitimise women’s candidacy for pelvic healthcare. This may require clinician education and training to better understand the significant effects of pelvic symptoms on women’s quality of life and wellbeing, to confidently educate women about their anatomy, their symptoms, and how to negotiate the healthcare system. Evidence informed, local pathways of care should be available and widely recognised to enable women to self-manage symptoms, when possible, to know when and where to seek help, and to expect to be supported by clinicians throughout their journey, with timely referral to specialist multidisciplinary services when required.

There are unanswered questions about facilitating early help-seeking in women with stigmatised pelvic symptoms: A few interventions have successfully increased pelvic health knowledge for a short duration [133–136], probably by improving cognitive representations of illness identity, but there is a lack of research targeting emotional representations to reduce the stigma of pelvic symptoms. Results from this systematic, mixed methods literature review suggest that changing stigma, knowledge, and beliefs about seeking help for pelvic symptoms will support women to identify their candidacy for healthcare, reduce normalising and deprioritising of symptoms, inappropriate self-help, and incorrect adjudication by clinicians who normalise and trivialise women’s pelvic symptoms. Future research needs to explore whether targeting both cognitive and emotional representations towards stigmatised pelvic symptoms, and the attitudes and norms women encounter, can encourage women to seek healthcare sooner. A successful intervention to raise awareness, reduce stigma and encourage women with stigmatised pelvic symptoms to seek timely healthcare could be used to better inform public health policy, reduce unnecessary surgical costs, and work towards meeting the United Nations Sustainable Development Goals core target 3.7 by 2030 [1].

### Electronic supplementary material

Below is the link to the electronic supplementary material.

Supplementary Material 1

Supplementary Material 2

Supplementary Material 3

Supplementary Material 4

## Abbreviations

CDSR: Cochrane Database of Systematic Reviews
CENTRAL: Cochrane Central Register of Controlled Trials
CSM: Common-Sense Model of Self-Regulation of Illness and Behaviour
FGM: Female Genital Mutilation
GRADE-CERQual: Grading of Recommendations Assessment, Development and Evaluation - Confidence in Evidence from Reviews of Qualitative research
HIV/AIDS: Human Immunodeficiency Virus/ Acquired Immunodeficiency Syndrome
HPV: Human Papilloma Virus
MMAT: Mixed Methods Appraisal Tool
PABAK: Prevalence And Bias Adjusted Kappa
PFD: Pelvic Floor Dysfunction
PRESS: Peer Review of Electronic Search Strategies
PRISMA: Preferred Reporting Items for Systematic Reviews and Meta-Analyses
SDG: Sustainable Development Goals
SPIDER: Sample, Phenomenon of Interest, Design, Evaluation, Research type, search structure
UI: Urinary Incontinence
UK: United Kingdom
UN: United Nations
US: United States
