# Supplementary material for: A mixed methods systematic literature review of barriers and facilitators to help-seeking among women with stigmatised pelvic health symptoms
Source: BMC Womens Health. 2024 Apr 3;24:217. doi: 10.1186/s12905-024-03063-6 (PMC10993589; doi:10.1186/s12905-024-03063-6)
Supplement: Supplementary file 1 — Supplementary Material 1 [file 12905_2024_3063_MOESM1_ESM.docx]

A mixed methods systematic literature review of barriers and facilitators to help-seeking among women with stigmatised pelvic health symptoms.

Clare Jouanny, University of Stirling, Faculty of Health Sciences and Sport, Stirling, Scotland (Corresponding author) [clj1@stir.ac.uk](mailto:clj1@stir.ac.uk)

ORCID: 0000-0002-4959-5901

Purva Abhyankar, University of Stirling, Department of Psychology, Stirling, Scotland

ORCID: 0000-0002-0779-6588

Margaret Maxwell, University of Stirling, The Nursing, Midwifery and Allied Health Professions Research Unit, Stirling, Scotland

ORCID: 0000-0003-3318-9500

**Additional File 1**

*Characteristics of quantitative, qualitative, and mixed methods studies*

| **Characteristics of Quantitative Studies** | | |  |  |  |  |
| --- | --- | --- | --- | --- | --- | --- |
| **Author(s) and**  **publication date** | **Symptom(s) studied** | **Country** | **Population (age: mean or range in years, ethnic/ clinical characteristics** | **Sample**  **size (n=)** | **Data collection method(s)** | **Help-seeking data collection in relation to overall study context** |
| Ahmed &  Fincham,  (2010) | Urinary tract infection (UTI) | USA | 49.5 yrs;  73.6% white, 22.3% black,  3% Asian, 6% Hispanic,  11% multi-racial  Clinical characteristics unknown | 493 | Telephone interview, at home (discrete choice experiment) | Discrete choice experiment with 8 choice scenarios for seeking care with acute UTI symptoms |
| Al-Badr et al. (2012) | Urinary incontinence | Saudi Arabia | 35 yrs;  76.% Saudi, 23.5% non-Saudi.  41.4% Urinary incontinence (UI);  36.4% Stress UI (SUI);  27.4% Urgency UI;  22.2% Mixed UI  3.7% Fecal incontinence | 374 | Questionnaire (in person, at primary health care centre) | Bespoke, piloted questionnaire with 6 parts: ‘behavior regarding medical advice’ part had 2 help seeking questions |
| Alshammari  et al. (2020) | Urinary incontinence | Saudi Arabia | 60 - >/=80 yrs;  Muslim;  41% mixed UI; | 78 | Interview and questionnaire (selfadministered at | Bespoke, expert reviewed, piloted, revised [face validity], 33 |

|  |  |  | 25.6% stress UI;  21.7% |  | public and private hospital out-patient clinics) | item questionnaire with 3 help seeking questions |
| --- | --- | --- | --- | --- | --- | --- |
| Alshenqeti et al. (2022) | Urinary incontinence | Saudi Arabia | 31.5 yrs;  92.7% Saudi, 7.3% non-Saudi;  Clinical characteristics unknown | 342 | Questionnaires (in- person at primary health care centres) | Bespoke questionnaire with unknow item total, with a choice of 5 reasons why they had not sought help |
| Berger et al. (2011) | Urinary incontinence | USA | 35-64 yrs;  53% black, 50.6% white.  60.1% had history of UTI; 44.3% obesity (BMI  >/= 30kg/m2); 63.2% current oestrogen use; 83.3% prior surgery for prolapse or UI; 57.6% prior hysterectomy. | 571 | Secondary data analysis of at home telephone interviews | Bespoke questionnaire with unknown item total, with 2 help seeking questions, including a choice of 6 reasons why they had not sought help |
| Choi et al. (2015) | Urinary incontinence | Korea | 30 - > 60 yrs;  Ethnicity and clinical characteristics unknown | 491 | Questionnaire  (telephone, at home) | Bespoke 16 item questionnaire with 3 help seeking questions |
| Cumming et al. (2010) | Urogenital atrophy | UK | unknown ages or ethnicity;  9% pre-menopausal, 33% peri-menopausal, 58% post-menopausal | 539 | Questionnaire (selfadministered online) | Bespoke 20 item questionnaire with unknown number of help seeking questions |
| Doshi et al. (2010) | Urinary incontinence in women with diabetes | USA | 54.7 yrs with diabetes, 56 years without diabetes;  White - 40% diabetic; 45.5% not diabetic;  Black - 21% diabetic; 19.1% not diabetic; Asian - 16.7% diabetic; 17.9% not diabetic;  Latina - 21.9% diabetic, 16.5% not diabetic;  Other - 0.4% diabetic, 0.9% not diabetic. | 602 | Questionnaires (selfadministered, at  home)  Interview (in-person) | Bespoke questionnaire with unknown number of items and help seeking questions |
| Dunivan et al. (2015) | Pelvic floor disorders (PFD) (urinary incontinence and prolapse) | USA | 77.7 yrs;  American Indian;  63% self reported UI; 20% self reported prolapse | 157 | Questionnaires (selfadministered before educational talk) | BICS-Q*: a reliable, validated 14item questionnaire that measures patient-reported barriers to care seeking for urinary incontinence |
| Elbiss,  Osman &  Hammad  (2013) | Urinary incontinence | United  Arab  Emirates  (UAE) | 37.8 yrs;  Emirati;  42.2% UI. | 429 | Questionnaire (in person, at family development centres) | Bespoke, piloted, modified, retested 28 item questionnaire with unknown number help seeking questions |
| Gambrah et al. (2022) | Sexual dysfunction (after pelvic fracture) | USA | 45.5 yrs;  White 87.8%; Asian 7.1%; Other 5.1% Clinical characteristics unknown | 98 | Questionnaires (selfadministered online) | Bespoke questionnaire with unknown number of items or help seeking questions |
| Gwee &  Setia (2012) | Chronic constipation | Singapore | 18 –70 yrs;  Ethnicity unknown; | 95 | Questionnaire (selfadministered online) | Bespoke 22 item questionnaire with 4 help seeking questions |

|  |  |  | Most common symptoms: 88% straining, 80% lumpy/hard stools |  |  |  |
| --- | --- | --- | --- | --- | --- | --- |
| Hammad,  Elbiss &  Osman  (2018) | Prolapse | UAE | 38.2 yrs;  Emirati;  100% prolapse symptoms | 127 | Questionnaire (in  person, at government  social/cultural centres) | Bespoke, piloted and retested questionnaire with 4 parts: one part was about help seeking |
| Hinchliff et al. (2020) | Sexual dysfunction | Norway,  Denmark,  Belgium,  Portugal | 60 - 75 yrs;  Ethnicity and clinical characteristics unknown | 1,940 | Questionnaire  (telephone, at home) | Bespoke 66 item questionnaire with 4 help seeking questions, including 8 potential choices for seeking, and 7 for not seeking help |
| Jarbol et al. (2021) | Urinary incontinence | Denmark | 20-80 yrs;  93.8% Danish, 6.2% immigrants;  Clinical characteristics unknown | 4,051 | Questionnaire (online or telephone at home) | Bespoke 5 item questionnaire with 2 help seeking questions |
| Krissi et al. (2012) | Urinary dysfunction or pelvic organ prolapse | Israel | 62.2 yrs;  Ethnicity and clinical characteristics unknown | 223 | Questionnaire (in person at  urogynaecology outpatient clinic of a tertiary medical center) | Bespoke questionnaire with unknown number of items or help seeking questions |
| Lamerton,  Mielke &  Brown  (2020) | Urinary incontinence | Australia | 24.7 yrs;  Ethnicity and clinical characteristics unknown | 986 asked about reasons for not seeking help out  of 8457 | Questionnaire (selfadministered online) | Standardized, pre-tested demographic and health behaviour questionnaires plus bespoke help seeking question with 14 potential reasons to chose why they had not sought help. |
| Mallett et al. (2018) | Urinary incontinence | USA | 18 – 66 yrs;  Mexican American, Hispanic, Chicana or Latina;  Clinical characteristics unknown | 209 | Questionnaire (in person at several tertiary care clinics, and at a community lecture on urinary incontinence) | BICS-Q* translated into Spanish by native speaker, plus 4 additional help seeking questions relevant to sample population. |
| Mann,  Shuster & | Pelvic pain | USA | 23 yrs;  78.6% white; 9.6% black or African American; | 390 | Questionnaire (selfadministered online) | Bespoke questionnaire with unknown number of items or |

| Moawad  (2013) |  |  | Clinical characteristics unknown |  |  | questions regarding barriers to care |
| --- | --- | --- | --- | --- | --- | --- |
| MoossdorffSteinhauser et al.  (2021a) | Urinary incontinence | The  Netherlands | 30.4yrs;  Ethnicity unknown;  100% pregnant: 35.9% nulliparous; 64.1% multiparous | 407 | Questionnaires (selfadministered online) | Reliable, validated symptom questionnaires, plus 4 bespoke, peer reviewed, piloted, modified and re-tested help seeking questions |
| MoossdorffSteinhauser et al.  (2021b) | Urinary incontinence | The  Netherlands | 30.6yrs;  Ethnicity unknown;  100% 6 weeks – 1 year post natal, 57.1% UI | 415 | Questionnaires (selfadministered online) | Reliable, validated symptom questionnaires, plus 4 bespoke, peer reviewed, piloted, modified and re-tested help seeking questions |
| Ng et al. (2014) | Urinary stress incontinence | Macau  SAR, China | 42.6yrs;  Ethnicity unknown;  37.5% SUI | 408 | Questionnaire (at Well Woman clinic, with help if needed) | Reliable, validated symptom questionnaires plus bespoke question to chose from 5 potential reasons if they had not sought help |
| Pakbaz et al. (2011) | Prolapse | Sweden | 51.7 yrs;  Ethnicity unknown;  38% prolapse 62% UI or awaiting hysterectomy (non-malignant) | 561 | Questionnaires (selfadministered online) | Bespoke, piloted, revised, retested questionnaire based on previous qualitative study; unknown number of items, including unknown number of questions regarding barriers to care |
| Po-Ming &  Chun-Hung (2021) | Urinary incontinence | Hong Kong | 43.6 yrs;  Ethnicity unknown;  66.4% UI | 639 | Questionnaires (selfadministered in clinic) | Bespoke questionnaire including a choice of 11 possible barriers to help seeking |
| Schreiber Pedersen et al. (2018) | Urinary incontinence | Germany and  Denmark | 50.3 yrs;  57.5% Danish, 42.5% German;  Clinical characteristics unknown | 1849 | Questionnaire (selfadministered at home | Reliable, validated symptom questionnaire with unknown number of bespoke, reliable, validated, questions about help seeking. |
| Smith et al. (2021) | Pelvic floor dysfunction | USA | 57.5 yrs;  Ethnicity and clinical characteristics unknown | 88 | Questionnaire (selfadministered at home) | Bespoke questionnaire including a choice of 10 possible barriers to help seeking |
| Tanaka et al. (2014) | Menstrual dysfunction | Japan | 34 yrs;  Ethnicity and clinical characteristics unknown | 774 | Questionnaires (selfadministered online) | Reliable, validated symptom questionnaire plus bespoke question to choose from 5 |
|  |  |  |  |  |  | potential reasons if they had not sought help |
| Tinetti et al. (2018) | Pelvic floor dysfunction | Australia | 68.6 yrs;  Ethnicity and clinical characteristics unknown | 376 | Questionnaires (selfadministered at home, or online) | Reliable, validated symptom questionnaire plus bespoke help  seeking questions adapted from BICS-Q* |
| Tudor et al. (2018) | Sexual dysfunction | UK | 42.4 yrs;  Ethnicity unknown;  100% Multiple Sclerosis, 29.6% bladder or urinary symptoms, 11.1% bowel symptoms, 5.4% gynaecological problems | 54 | Questionnaire (selfcompleted at outpatient clinic, or MS Centre) | Bespoke, reviewed and amended, 29-item questionnaire including a choice of possible barriers to help seeking |
| Waetjen et al. (2018) | Urinary incontinence | USA | 42 -52 yrs;  White, African American, Asian;  100% at menopausal transition | 814 | Questionnaire (selfadministered at home) | Bespoke questionnaire based on  The Common Sense Model of Illness Representations with 7 reasons condensed into 3 potential categories for why they had not sought help |
| Washington et al. (2013) | Pelvic floor dysfunction | USA | 57 yrs;  African American;  Clinical characteristics unknown | 362 | Questionnaire (selfadministered during a conference) | Bespoke questionnaire with 4 domains: one included help  seeking questions adapted from BICS-Q* |
| Willis-Gray et al. (2015) | Urinary incontinence | USA | 46.3 yrs;  White, black, Latina;  Clinical characteristics unknown | 93 | Questionnaires (selfadministered in  clinics and at community groups) | Reliable, validated symptom questionnaires plus BICS-Q* translated into Spanish by certified healthcare translation service |
| Wojtowicz  et al. (2014) | Urinary incontinence | Poland | 59.9 yrs;  Ethnicity and clinical characteristics unknown | 141 | Questionnaire (selfadministered in clinic or | Polish version of a validated symptom questionnaire with unknown number of bespoke help seeking questions |

**Characteristics of Qualitative and Mixed Methods Studies**

**Author(s) and Symptom(s) Country** **Population (age: mean or range in years; ethnic/ Sample size Theoretical Data collection**

**publication date** **studied** **clinical characteristics; context)** **(n=)** **framework method(s)**

| Abhyankar et al. (2019) | Prolapse | UK | Age: unknown  21 Caucasian, 1 Asian; receiving prolapse care in  NHS urogynaecology clinics | 22 | Symbolic interactionism | Focus groups and semistructured interviews |
| --- | --- | --- | --- | --- | --- | --- |
| Bascur-Castillo et al. (2019) | Urinary incontinence | Chile | 33 – 53 yrs; UI diagnosed at tertiary hospital | 10 | The Anderson Model | Semi-structured interviews |
| Beaumont et al. (2022) | Pelvic floor dysfunction  (PFD) | Australia | 35 – 73 yrs;  Ethnicity unknown; all women had prolapse, urinary incontinence, anal incontinence or a combination. 5 women had gynaecological appointments indefinitely postponed due to Covid19 pandemic | 11 | Not stated | Semi-structured interviews |
| Bjork et al. (2014) | Stress Urinary incontinence (SUI) | Sweden | 47.6 yrs;  Rural and urban Swedish;  Participated in RCT* comparing PFMT** delivery | 21 | Not stated | Semi-structured interviews |
| Brown, Rogers & Wise (2017) | Anal incontinence (AI) | USA | 46 – 85 yrs;  89 % white, 8 % African American, 3 % Latina;  Self-reported AI in last 3 months | 39 | Not stated | Focus groups and semistructured interviews |
| Buurman &  Lagro-Janssen (2013) | PFD | The  Netherlands | 20 – 40 yrs;  24 Dutch, 1 Indonesian, 1 Bulgarian; Post natal | 26 | Not stated | Semi-structured interviews |
| Carroll et al. (2022) | Prolapse | UK | 36.8 yrs;  Ethnicity unknown;  5 -Prolapse stage I, 6 -stage II, 3 -stage III; 3 using a pessary | 14 | Realist  phenomeno-  logy | Semi-structured interviews |
| Carsughi, Santini & Lamura (2019) | Urinary incontinence | Italy | 75.2 yrs; Ethnicity unknown;  Different UI severity on admission to Urology and Neurology wards | 6 | Not stated | Semi-structured interviews |
| Chen et al. (2018) | Pelvic pain | USA | 33.6 yrs;  76.1% Caucasian;  10.8% had diagnosed conditions contributory to pelvic pain, 69.2% had another chronic pain condition | 509 | Not stated | Questionnaire with one open-ended question about why they did not seek care |
| Chen et al. (2020) | Interstitial cystitis (IC)/ Painful bladder syndrome | Taiwan | 53 yrs; Ethnicity unknown;  75% had symptoms < 5 yrs, 98.5% had nonulcerative IC; | 22 | Not stated | Semi-structured interviews |
| Cross et al. (2014) | Urinary and fecal incontinence | Australia | < 65 yrs; | 167 | Not stated | Focus groups |

|  |  |  | Culturally and linguistically diverse groups: Arabic,  Chinese, Greek, Italian, Macedonian, Polish,  Russian, Spanish-speaking, Turkish, Vietnamese |  |  |  |
| --- | --- | --- | --- | --- | --- | --- |
| Devendorf et al. (2020) | Urinary and fecal incontinence | USA | 89% were >50 yrs;  Veterans; Ethnicity unknown;  All had UI, some also had fecal incontinence | 9 | Stigma framework (for analysis only) | Semi-structured interviews |
| Donaldson &  Meana (2011) | Pelvic pain  (dyspareunia) | USA | 19.07 years;  9 European-American, 3 African-American, 1  Hispanic, 1 Asian-American;  Persistent pain for an average of 15 months | 14 | Not stated | Semi-structured interviews |
| Fileborn et al. (2017) | Sexual dysfunction | Australia | 55 – 79 years;  Ethnicity, clinical characteristics unknown Part of a larger national study on sex and sexual health | 23 | Not stated | Semi-structured interviews |
| Ghetti et al. (2015) | Prolapse | USA | 60 years;  89% Caucasian;  Prolapse diagnosis was based on physical examination; 28 were post surgery, 4 awaiting surgery; 9 conservative treatment. | 43 | Not stated | Focus groups and semistructured interviews |
| Gonzalez et al. (2019) | SUI | Worldwide  (online) | Age, ethnicity, clinical characteristics: unknown. | 200 randomly selected posts from 762 unique users | The Social Ecological model | Digital ethnography |
| Gore-Gorszewska (2020) | Sexual dysfunction | Poland | 65 – 82 years;  100% Polish;  Self-selected through public posters | 16 | Not stated | Semi-structured interviews |
| Grundstrom et al. (2018) | Pelvic pain  (endometriosis) | Sweden | 37 years (median);  Ethnicity unknown  Laparoscopically diagnosed endometriosis 1 -34 years | 9 | Not stated | Semi-structured interviews |
| Grundstrom et al. (2020) | Pelvic pain  (endometriosis) | Sweden | 28 years;  Ethnicity unknown  Endometriosis diagnosis = 1-7 years | 16 blogs | Not stated | Digital ethnography |
| Hatchett et al. (2011) | PFD | USA | 40.1 years (African American)  41.8 years (Latin American);  46.9% African American, 53.1% Latin American. | 32 | Not stated | Focus groups |

| Hayder (2012) | Sexual dysfunction | Germany | Age: unknown for women only.  Ethnicity unknown;  Lived with UI for 18 months – 35 years | 22 | Not stated | Problem-centred interviews |
| --- | --- | --- | --- | --- | --- | --- |
| Hinchliff et al. (2018) | Sexual dysfunction | UK | <50 years - >90 years;  Ethnicity unknown;  57.8% had engaged in sexual activity in the last year | 668 | Not stated | Open ended question as part of larger survey study |
| Jackson et al. (2012) | Urinary dysfunction | USA | 30 – 79 years;  34.2% Black, 34.2% Hispanic, 31.5% White;  Sub-set of a larger study, with one or more urinary symptoms | 73 | Not stated | Structured interviews |
| Jackson et al. (2017) | Pelvic floor dysfunction (prolapse and UI) | USA | 51.8 years;  100% Hispanic;  Clinical characteristics unknown | 24 | Not stated | Focus groups |
| Jurgensen et al. (2015) | Urinary incontinence | Germany | 41 – 86 years;  Ethnicity unknown;  Self reported UI symptoms | 49 | Not stated | Focus groups |
| Leusink et al. (2019) | Pelvic pain (vulvodynia) | The  Netherlands | 20 – 50 years;  Ethnicity unknown;  3 had chronic pain, 5 had anxiety/mood disorder, 2 had PF overactivity, 2 had no medical/psychological history | 12 | Not stated | Semi-structured interviews |
| Low &  Tumbarello  (2012) | Prolapse | USA | 57.4 years;  100% white;  9 had not had prolapse surgery, 5 had surgery | 13 | Analytical  Framework of  Authoritative  Knowledge | Semi-structured interviews |
| Milner, Gamble  & Barry-Kinsella  (2022) | PFD | Worldwide  (online) | Age and ethnicity unknown;  41% bladder control problems; 37% prolapse; 6% pelvic pain; 4% sexual dysfunction; 3% faecal incontinence | 70 | Not stated | Open ended question as part of larger survey study |
| Milroy, Jacobs & Frayne (2022) | PFD | Australia | 50.3 yrs;  Ethnicity and clinical characteristics unknown | 27 | Not stated | Semi-structured interviews |
| Mirskaya,  Lindgren &  Carlsson (2019) | Prolapse | Sweden | 27 years (for 17 whose age was identifiable);  Ethnicity and clinical characteristics unknown | 33 | Not stated | Digital ethnography |
| Moossdorff-  Steinhauser et al.  (2023) | UI (peri-partum) | The  Netherlands | 24-38 yrs;  Ethnicity unknown;  6 pregnant women, 7 post partum women | 13 | Not stated | Focus groups and semistructured interviews |

| Newton et al. (2013) | Pelvic pain (pelvic inflammatory disease) | Australia | 28 years;  65.2% Australian born;  86.9% less than 4 months post-diagnosis | 23 | Not stated | Semi-structured interviews |
| --- | --- | --- | --- | --- | --- | --- |
| Pakbaz et al. (2010) | Prolapse | Sweden | 56.5 years;  Ethnicity unknown;  Awaiting prolapse surgery, with self-reported vaginal prolapse symptoms for 2-10 years | 14 | Not stated | Semi-structured interviews |
| Pintos-Diaz et al. (2019) | UI | Spain | 47.3 years;  Ethnicity unknown;  First attendance at specialist UI centre with 16.6% mixed UI, 61.1% stress UI, 22.2% urge UI, for 1 – 46 months | 18 | Not stated | Semi-structured interviews and personal  letters |
| Roin & Nord (2015) | Urinary incontinence | Faroe Islands (Denmark) | 60 – 65 years;  Ethnicity unknown;  UI restricted daily lives | 7 | Not stated | Semi-structured interviews |
| Schaller, Traeen  &Lundin Kvalem  (2020) | Sexual dysfunction | Norway | 65 – 85 years;  100% Norwegian; | 15 | Not stated | Semi-structured interviews |
| Siddiqui et al. (2016) | UI | USA | 44.5 years;  34.5% White, 36.3% Black, 29.2% Latina;  Self reported daily, weekly or monthly UI | 113 | Not stated | Focus groups |
| Siu (2015) | Urinary dysfunction (overactive bladder) | Hong Kong | 28-55 years;  100% Hong Kong Chinese;  Members of an OAB self-help group, symptomatic  5-11years, diagnosed 1-6 years | 30 | Not stated | Semi-structured interviews |
| Tucker et al. (2019) | Anal incontinence (AI) | Australia | 31 years;  81% Caucasian, 19% Aboriginal; Pregnant, with previous history of AI | 16 | Not stated | Semi-structured interviews |
| TuiSamoa,  Heather & Kruger  (2022) | UI | New Zealand | 23-63 yrs;  6 Samoan, 3 Tongan, 1 Cook Islander; 2 had no experience of UI, some had cared for women with UI; 3 nurses, 2 allied health professionals, 5 administrators  Clinical characteristics unknown | 10 | Not stated | Focus group |
| Vardeman, Spiers  & Yamasaki  (2022) | PFD | USA | 23 – 82 yrs; | 22 | Narrative theorising | Semi-structured interviews |

|  |  |  | 14 White, 4 Black, 3 Latina, 1 Asian; all reported varying severity of PFD but one had lived experience of caring for mother with PFD |  |  |  |
| --- | --- | --- | --- | --- | --- | --- |
| Vethanayagam et al. (2017) | UI | UK | 67.4 years;  Majority White British;  UI daily or several times a week that interfered with daily life | 50 | Not stated | Semi-structured interviews |
| Wagg, Kendall & Bunn (2017) | Urinary dysfunction | UK | 23-41 years;  Ethnicity unknown;  Post-partum, from one general practice | 15 | Not stated | Semi-structured interviews |
| Wang et al. (2011) | Urinary dysfunction | Taiwan | 53 years;  100% Taiwanese;  11=UI only; 3=daytime frequency & nocturia; 1=UI, nocturia & urgency; 1=UI, feeling of incomplete emptying; Symptomatic for 6 -20yrs | 16 | Not stated | Semi-structured interviews |
| Welch, Botelho & Tennstedt (2011) | Urinary dysfunction | USA | Age: unknown for women only;  Black, Hispanic and white;    Subset of larger study, reporting at least one urinary symptom | 17 | Shaw’s  Framework for the Study of Coping, Illness behaviour and Outcomes | Semi-structured interviews |
| Welch,  Taubenberger &  Tennstedt (2011) | Urinary dysfunction | USA | 57.2 years (Black), 62.7 years (Hispanic), 58.9 years (White);  34.1% Black, 34.1% Hispanic, 31.7% White; Subset of a larger study, reporting UI, frequency, nocturia, urgency, incomplete emptying | 41 | Not stated | Semi-structured interviews |
| Wieslander et al. (2015) | Prolapse | USA | 63.8 years (English speakers), 56.6 years (Spanish speakers);  83% American (English speakers), 70% Mexican  (Spanish speakers);  Prolapse diagnosed on physical examination | 58 | Not stated | Focus groups |
| Young, Fisher & Kirkman (2019) | Pelvic pain  (endometriosis) | Australia | 34.4 years;  80.8% Australian, 3.8% South African, 3.8% East  Timor, 3.8% Hong Kong, 3.8% French, 3.8% SriLankan;  Average time since diagnosis 3.5 years | 26 | Feminist Social Constructionist framework | Semi-structured interviews |
| **Mixed methods** |  |  |  |  |  |  |
| Drennan et al. (2010) | Bladder and bowel dysfunction | UK | <20 - >50 years;  Ethnicity unknown;  Female prisoners; 50% taking daily prescribed medication for mental/physical ill health | 148 survey; 46 open-ended responses | Not stated | Bespoke, validated, piloted self-report questionnaire with some open ended questions |
| Mapp et al. (2019) | Genitourinary symptoms | UK | 16 – 44 years (questionnaires), 19 – 47 years  (interviews);  White, Asian, Black;  Sub-set from National Survey of Sexual Attitudes and Lifestyles study | 1182 survey;  16 interviews | Not stated | Validated questionnaire; semi-structured interviews |
| Muller (2010) | Prolapse | USA | 64 years (weighted average age);  93.8% white/Caucasian;  Self reported: 67.5% UI and prolapse, 23.8% on prescribed medication for bladder control, 67.5% anterior wall prolapse/ cystocele, 33.8% posterior wall prolapse/rectocele, 37.6% multiple vaginal prolapse | 97 survey; 33 interviews | Not stated | Bespoke questionnaire with unknown number of questions about help seeking; semi-structured interviews |
| O’Malley, Smith & Higgins (2021) | Sexual dysfunction | Ireland | 22 – 43 yrs;  Ethnicity unknown; post-partum women, the age of the baby was mean 27 months; 47% had a second baby at time of interview. | 1,408 survey;  21 interviews | Not stated | Published, modified, piloted questionnaires; semi-structured interviews |
| Rutte et al. (2016) | Sexual dysfunction | The  Netherlands | 58.5 years (questionnaires), 61.9 years (interviews);  Ethnicity unknown;  100% Type 2 Diabetes | 48 survey; 11 interviews | Not stated | Published self- report questionnaires; semistructured interviews |

*RCT – randomised controlled trial **PFMT – pelvic floor muscle training
