# Supplementary material for: A mixed methods systematic literature review of barriers and facilitators to help-seeking among women with stigmatised pelvic health symptoms
Source: BMC Womens Health. 2024 Apr 3;24:217. doi: 10.1186/s12905-024-03063-6 (PMC10993589; doi:10.1186/s12905-024-03063-6)
Supplement: Supplementary file 2 — Supplementary Material 2 [file 12905_2024_3063_MOESM2_ESM.docx]

A mixed methods systematic literature review of barriers and facilitators to help-seeking among women with stigmatised pelvic health symptoms.

Clare Jouanny, University of Stirling, Faculty of Health Sciences and Sport, Stirling, Scotland (Corresponding author) clj1@stir.ac.uk

ORCID: 0000-0002-4959-5901

Purva Abhyankar, University of Stirling, Department of Psychology, Stirling, Scotland

ORCID: 0000-0002-0779-6588

Margaret Maxwell, University of Stirling, The Nursing, Midwifery and Allied Health Professions Research Unit, Stirling, Scotland

ORCID: 0000-0003-3318-9500

**Additional File 2**

*Quality assessment of all studies using the MMAT*

| **Author(s) and publication date** | **All studies** | | **Qualitative** | | | | | **Quantitative descriptive** | | | | | **Mixed methods** | | | |  |
| --- | --- | --- | --- | --- | --- | --- | --- | --- | --- | --- | --- | --- | --- | --- | --- | --- | --- |
| **QUANTITATIVE** | **S1** | **S2** | **1.1** | **1.2** | **1.3** | **1.4** | **1.5** | **4.1** | **4.2** | **4.3** | **4.4** | **4.5** | **5.1** | **5.2** | **5.3** | **5.4** | **5.5** |
| Ahmed & Fincham, (2010) | Y | Y |  |  |  |  |  | Y | N | Y | ? | Y |  |  |  |  |  |
| Al-Badr et al. (2012) | Y | Y |  |  |  |  |  | Y | Y | Y | Y | Y |  |  |  |  |  |
| Alshammari et al. (2020) | Y | Y |  |  |  |  |  | Y | Y | Y | ? | Y |  |  |  |  |  |
| Alshenqeti et al. (2022) | Y | Y |  |  |  |  |  | Y | Y | Y | ? | N |  |  |  |  |  |
| Berger et al. (2011) | Y | Y |  |  |  |  |  | Y | ? | Y | Y | Y |  |  |  |  |  |
| Choi et al. (2015) | Y | Y |  |  |  |  |  | Y | ? | ? | ? | Y |  |  |  |  |  |
| Cumming et al. (2010) | Y | Y |  |  |  |  |  | Y | ? | ? | ? | Y |  |  |  |  |  |
| Doshi et al. (2010) | Y | Y |  |  |  |  |  | Y | ? | ? | ? | Y |  |  |  |  |  |
| Dunivan et al. (2015) | Y | Y |  |  |  |  |  | Y | Y | Y | ? | Y |  |  |  |  |  |
| Elbiss, et al. (2013) | Y | Y |  |  |  |  |  | Y | Y | ? | ? | Y |  |  |  |  |  |
| Gambrah et al. (2022) | Y | Y |  |  |  |  |  | Y | ? | ? | N | Y |  |  |  |  |  |
| Gwee & Setia (2012) | Y | Y |  |  |  |  |  | Y | Y | Y | ? | Y |  |  |  |  |  |
| Hammad et al. (2018) | Y | Y |  |  |  |  |  | ? | Y | ? | ? | Y |  |  |  |  |  |
| Hinchliff et al. (2020) | Y | Y |  |  |  |  |  | Y | Y | Y | ? | Y |  |  |  |  |  |

| Jarbol et al. (2021) | Y | Y |  |  |  |  |  | ? | Y | Y | ? | Y |  |  |  |  |  |
| --- | --- | --- | --- | --- | --- | --- | --- | --- | --- | --- | --- | --- | --- | --- | --- | --- | --- |
| Krissi et al. (2012) | Y | Y |  |  |  |  |  | ? | ? | ? | ? | Y |  |  |  |  |  |
| Lamerton et al. (2020) | Y | Y |  |  |  |  |  | ? | Y | Y | ? | Y |  |  |  |  |  |
| Mallett et al. (2018) | Y | Y |  |  |  |  |  | Y | ? | Y | ? | Y |  |  |  |  |  |
| Mann et al. (2013) | Y | Y |  |  |  |  |  | Y | N | ? | N | Y |  |  |  |  |  |
| Moossdorff-  Steinhauser et al.  (2021a) | Y | Y |  |  |  |  |  | Y | Y | Y | ? | Y |  |  |  |  |  |
| Moossdorff-  Steinhauser et al.  (2021b) | Y | Y |  |  |  |  |  | Y | ? | Y | ? | Y |  |  |  |  |  |
| Ng et al. (2014) | Y | Y |  |  |  |  |  | Y | ? | Y | ? | Y |  |  |  |  |  |
| Pakbaz et al. (2011) | Y | Y |  |  |  |  |  | ? | ? | Y | ? | Y |  |  |  |  |  |
| Po-Ming & ChunHun (2021) | Y | Y |  |  |  |  |  | Y | ? | Y | N | Y |  |  |  |  |  |
| Schreiber Pedersen et al. (2018) | Y | ? |  |  |  |  |  | Y | Y | Y | ? | Y |  |  |  |  |  |
| Smith et al. (2021) | Y | Y |  |  |  |  |  | Y | Y | ? | Y | Y |  |  |  |  |  |
| Tanaka et al. (2014) | Y | Y |  |  |  |  |  | Y | Y | Y | ? | Y |  |  |  |  |  |
| Tinetti et al. (2018) | Y | Y |  |  |  |  |  | ? | ? | Y | ? | Y |  |  |  |  |  |
| Tudor et al. (2018) | Y | Y |  |  |  |  |  | Y | ? | Y | ? | Y |  |  |  |  |  |
| Waetjen et al. (2018) | Y | Y |  |  |  |  |  | Y | ? | Y | ? | Y |  |  |  |  |  |
| Washington et al. (2013) | Y | Y |  |  |  |  |  | Y | ? | Y | ? | Y |  |  |  |  |  |
| Willis-Gray et al. (2015) | Y | Y |  |  |  |  |  | ? | ? | Y | ? | Y |  |  |  |  |  |
| Wojtowicz et al. (2014) | Y | Y |  |  |  |  |  | ? | ? | ? | ? | Y |  |  |  |  |  |
| **QUALITATIVE** |  |  |  |  |  |  |  |  |  |  |  |  |  |  |  |  |  |
| Abhyankar et al. (2019) | Y | Y | Y | Y | Y | Y | Y |  |  |  |  |  |  |  |  |  |  |
| Bascur-Castillo et al. (2019) | Y | Y | Y | Y | Y | Y | Y |  |  |  |  |  |  |  |  |  |  |
| Beaumont et al. (2022) | Y | Y | Y | Y | Y | Y | Y |  |  |  |  |  |  |  |  |  |  |
| Bjork et al. (2014) | Y | Y | Y | Y | ? | N | Y |  |  |  |  |  |  |  |  |  |  |
| Brown et al. (2017) | Y | Y | Y | Y | Y | Y | Y |  |  |  |  |  |  |  |  |  |  |
| Buurman & Lagro-  Janssen (2013) | Y | Y | Y | Y | Y | Y | Y |  |  |  |  |  |  |  |  |  |  |
| Carroll et al. (2022) | Y | Y | Y | Y | Y | Y | Y |  |  |  |  |  |  |  |  |  |  |
| Carsughi et al. (2019) | Y | Y | Y | Y | ? | N | ? |  |  |  |  |  |  |  |  |  |  |
| Chen et al. (2018) | Y | ? | Y | Y | ? | ? | ? |  |  |  |  |  |  |  |  |  |  |
| Chen et al. (2020) | Y | ? | Y | N | N | N | N |  |  |  |  |  |  |  |  |  |  |
| Cross et al. (2014) | Y | Y | Y | Y | Y | Y | ? |  |  |  |  |  |  |  |  |  |  |
| Devendorf et al. (2020) | Y | Y | Y | Y | Y | N | ? |  |  |  |  |  |  |  |  |  |  |

| Donaldson & Meana (2011) | Y | Y | Y | Y | Y | Y | Y |  |  |  |  |  |  |  |  |  |  |
| --- | --- | --- | --- | --- | --- | --- | --- | --- | --- | --- | --- | --- | --- | --- | --- | --- | --- |
| Fileborn et al. (2017) | Y | Y | Y | Y | Y | Y | Y |  |  |  |  |  |  |  |  |  |  |
| Ghetti et al. (2015) | Y | Y | Y | Y | Y | Y | Y |  |  |  |  |  |  |  |  |  |  |
| Gonzalez et al. (2019) | Y | ? | Y | Y | ? | ? | ? |  |  |  |  |  |  |  |  |  |  |
| Gore-Gorszewska (2020) | Y | Y | Y | Y | Y | Y | Y |  |  |  |  |  |  |  |  |  |  |
| Grundstrom et al. (2018) | Y | Y | Y | Y | Y | Y | Y |  |  |  |  |  |  |  |  |  |  |
| Grundstrom et al. (2020) | Y | Y | Y | Y | Y | Y | Y |  |  |  |  |  |  |  |  |  |  |
| Hatchett et al. (2011) | Y | Y | Y | Y | Y | Y | Y |  |  |  |  |  |  |  |  |  |  |
| Hayder (2012) | Y | Y | Y | Y | Y | Y | Y |  |  |  |  |  |  |  |  |  |  |
| Hinchliff et al. (2018) | Y | Y | Y | Y | Y | Y | Y |  |  |  |  |  |  |  |  |  |  |
| Jackson et al. (2012) | Y | Y | Y | Y | Y | Y | Y |  |  |  |  |  |  |  |  |  |  |
| Jackson et al. (2017) | Y | Y | Y | Y | Y | Y | Y |  |  |  |  |  |  |  |  |  |  |
| Jurgensen et al. (2015) | Y | Y | Y | Y | Y | Y | Y |  |  |  |  |  |  |  |  |  |  |
| Leusink et al. (2019) | Y | Y | Y | Y | Y | Y | Y |  |  |  |  |  |  |  |  |  |  |
| Low & Tumbarello (2012) | Y | Y | Y | Y | Y | Y | ? |  |  |  |  |  |  |  |  |  |  |
| Milner et al. (2022) | Y | Y | Y | ? | Y | Y | Y |  |  |  |  |  |  |  |  |  |  |
| Milroy et al. (2022) | Y | Y | Y | Y | Y | Y | Y |  |  |  |  |  |  |  |  |  |  |
| Mirskaya et al. (2019) | Y | Y | Y | Y | Y | Y | Y |  |  |  |  |  |  |  |  |  |  |
| Moossdorff-  Steinhauser et al.  (2023) | Y | Y | Y | Y | Y | Y | Y |  |  |  |  |  |  |  |  |  |  |
| Newton et al. (2013) | Y | Y | Y | Y | Y | Y | Y |  |  |  |  |  |  |  |  |  |  |
| Pakbaz et al. (2010) | Y | Y | Y | Y | Y | Y | Y |  |  |  |  |  |  |  |  |  |  |
| Pintos-Diaz et al. (2019) | Y | Y | Y | Y | Y | Y | Y |  |  |  |  |  |  |  |  |  |  |
| Roin & Nord (2015) | Y | Y | Y | Y | Y | Y | Y |  |  |  |  |  |  |  |  |  |  |
| Schaller et al. (2020) | Y | Y | Y | Y | Y | Y | Y |  |  |  |  |  |  |  |  |  |  |
| Siddiqui et al. (2016) | Y | Y | Y | Y | Y | Y | ? |  |  |  |  |  |  |  |  |  |  |
| Siu (2015) | Y | Y | Y | Y | Y | Y | Y |  |  |  |  |  |  |  |  |  |  |
| Tucker et al. (2019) | Y | Y | Y | Y | Y | Y | Y |  |  |  |  |  |  |  |  |  |  |
| TuiSamoa et al. (2022) | Y | Y | Y | Y | Y | Y | Y |  |  |  |  |  |  |  |  |  |  |
| Vardeman et al. (2022) | Y | Y | Y | Y | Y | Y | Y |  |  |  |  |  |  |  |  |  |  |
| Vethanayagam et al. (2017) | Y | Y | Y | Y | Y | Y | Y |  |  |  |  |  |  |  |  |  |  |
| Wagg et al. (2017) | Y | Y | Y | Y | Y | Y | Y |  |  |  |  |  |  |  |  |  |  |
| Wang et al. (2011) | Y | Y | Y | Y | Y | Y | Y |  |  |  |  |  |  |  |  |  |  |
| Welch et al. (2011a) | Y | Y | Y | Y | Y | Y | Y |  |  |  |  |  |  |  |  |  |  |
| Welch et al. (2011b) | Y | Y | Y | Y | Y | Y | Y |  |  |  |  |  |  |  |  |  |  |
| Wieslander et al. (2015) | Y | Y | Y | Y | Y | Y | ? |  |  |  |  |  |  |  |  |  |  |
| Young et al. (2019) | Y | Y | Y | Y | Y | Y | Y |  |  |  |  |  |  |  |  |  |  |
| **MIXED**  **METHODS** |  |  |  |  |  |  |  |  |  |  |  |  |  |  |  |  |  |
| Drennan et al. (2010) | Y | Y | Y | Y | Y | Y | Y | Y | N | Y | ? | Y | Y | Y | Y | Y | ? |
| Mapp et al. (2019) | Y | Y | Y | Y | Y | Y | Y | Y | Y | Y | ? | Y | Y | Y | Y | Y | Y |
| Muller (2010) | Y | Y | Y | Y | Y | Y | Y | Y | ? | ? | N | Y | Y | N | ? | ? | ? |
| O’Malley et al. (2021) | Y | Y | Y | Y | Y | Y | Y | Y | N | Y | Y | Y | Y | Y | Y | Y | Y |
| Rutte et al. (2016) | Y | Y | Y | Y | Y | Y | Y | ? | N | Y | N | Y | Y | Y | Y | ? | Y |
