# Supplementary material for: A mixed methods systematic literature review of barriers and facilitators to help-seeking among women with stigmatised pelvic health symptoms
Source: BMC Womens Health. 2024 Apr 3;24:217. doi: 10.1186/s12905-024-03063-6 (PMC10993589; doi:10.1186/s12905-024-03063-6)
Supplement: Supplementary file 3 — Supplementary Material 3 [file 12905_2024_3063_MOESM3_ESM.docx]

A mixed methods systematic literature review of barriers and facilitators to help-seeking among women with stigmatised pelvic health symptoms.

Clare Jouanny, University of Stirling, Faculty of Health Sciences and Sport, Stirling, Scotland (Corresponding author) clj1@stir.ac.uk ORCID: 0000-0002-4959-5901

Purva Abhyankar, University of Stirling, Department of Psychology, Stirling, Scotland

ORCID: 0000-0002-0779-6588

Margaret Maxwell, University of Stirling, The Nursing, Midwifery and Allied Health Professions Research Unit, Stirling, Scotland

ORCID: 0000-0003-3318-9500

**Additional File 3**

*Papers referencing the key barriers and facilitators to seeking help with stigmatised pelvic symptoms*

| **BARRIERS** | No. of quantitative papers citing barrier | References | No. of qualitative/ mixed methods  papers citing barrier | References |
| --- | --- | --- | --- | --- |
| Stigma  (embarrassment, shame, taboo) | 22 | Al-Badr et al., (2012); Alshammari et al., (2020); Alshenqeti et al., (2022); Berger et al., (2011); Choi et al., (2015); Cumming et al., (2010); Elbiss et al., (2013); Gambrah et al., (2022); Gwee and Setia, (2012); Hammad et al., (2018); Hinchliff et al.,  (2020); Jarbøl et al., (2021); Lamerton et al.,  (2020); Mallett et al., (2018); Ng et al.,  (2014); Pakbaz et al., (2011); Po-ming & Chun-Hung, (2021); Schreiber Pedersen et al., (2018); Smith et al., (2021); Tinetti et al., (2018); Tudor et al., (2018); Wójtowicz et al., (2014) | 30 | Abhyankar et al., (2019); Bjork et al., (2014); Brown,  Rogers & Wise (2017); Buurman & Lagro-Janssen,  (2013); Carroll et al., (2022); Cross et al., (2014);  Donaldson & Meana, (2011); Drennan et al., (2010);  Fileborn et al., (2017); Ghetti et al., (2015); GoreGorszewska (2020); Grundstrom et al., (2020); Hatchett et al., (2011); Hayder (2012); Jackson et al., (2017); Jurgensen et al., (2015); Leusink et al., (2019); Mapp et al., (2019); Milroy, Jacobs & Frayne, (2022); Pakbaz et al., (2010); Pintos-Diaz et al., (2019); Roin & Nord,  (2015); Schaller, Traeen &Lundin Kvalem, (2020);  Siddiqui et al., (2016); Siu, (2015); Tucker et al., (2019);  TuiSamoa, Heather & Kruger, (2022); Vardeman, Spiers & Yamasalu (2022); Wagg, Kendall & Bunn, (2017); Wang et al., (2011). |

| Lack of knowledge:      -General (where to seek help/ treatment options)            -Hoping for spontaneous symptom resolution | 18                10 | Al-Badr et al., (2012); Alshammari et al., (2020); Alshenqeti et al., (2022); Berger et al., (2011); Cumming et al., (2010); Doshi et al., (2010); Elbiss et al., (2013); Gambrah et al., (2022); Hammad et al., (2018); Krissi et al., (2012); Pakbaz et al., (2011); Tinetti et al., (2018); Tudor et al., (2018); Po-ming &  Chun-Hung, (2021); Smith et al., (2021);  Tanaka et al., (2014); Hinchliff et al.,  (2020); Washington et al., (2013)    Alshammari et al., (2021); Elbiss et al.,  (2013); Gambrah et al., (2022); Gwee and  Setia, (2012); Hammad et al., (2018);  Hinchliff et al., (2020); Lamerton et al.,  (2020),;Moossdorrf-Steinhauser et al.,  (2021a, 2021b); Wójtowicz et al., (2014) | 23                  0 | Bascur-Castillo et al. (2019); Brown, Rogers & Wise  (2017); Buurman & Lagro-Janssen (2013); Carroll et al., 2022; Chen et al. (2018); Donaldson & Meana (2011); Drennan et al. (2010); Ghetti et al. (2015); Jackson et al.  (2017); Jurgensen et al. (2015); Leusink et al. (2019);  Mapp et al. (2019); Mirskaya, Lindgren & Carlsson (2019); Muller (2010); Newton et al. (2013); Pakbaz et al. (2010); Pintos-Diaz et al. (2019); Roin & Nord (2015); Rutte et al. (2016); Siddiqui et al. (2016); Tucker et al. (2019); TuiSamoa, Heather & Kruger, 2022;  Wieslander et al. (2015)      - |
| --- | --- | --- | --- | --- |
| -Deprioritising | 19 | Al-Badr et al., (2012); Alshammari et al.,  (2020); Alshenqeti et al., (2022); Choi et al.,  (2015); Gambrah et al., (2022); Gwee and Setia, (2012); Hinchliff et al., (2020); Krissi et al., (2012); Lamerton et al., (2020); Moossdorff-Steinhauser et al., (2021a,  2021b); Pakbaz et al., (2011); Po-ming &  Chun-Hung, (2021); Schreiber Pedersen et al., (2018); Tanaka et al., (2014); Tinetti et al., (2018); Tudor et al., (2018); Waetjen et al., (2018); Wójtowicz et al., (2014) | 19 | Abhyankar et al. (2019); Bascur-Castillo et al. (2019);  Brown, Rogers & Wise (2017); Buurman & LagroJanssen (2013); Carsughi, Santini & Lamura (2019);  Chen et al. (2018); Devendorf et al. (2020); Donaldson & Meana (2011); Hatchett et al. (2011); Jackson et al. (2017); Jurgensen et al. (2015); Leusink et al. (2019);  Mapp et al. (2019); Milroy, Jacobs & Frayne, (2022);  Pakbaz et al. (2010); Siddiqui et al. (2016); Siu (2015);  Wagg, Kendall & Bunn (2017); Welch, Botelho &  Tennstedt (2011) |
| -Normalising | 15 | Al-Badr et al., (2012); Alshammari et al.,  (2020); Alshenqeti et al., (2022); Cumming et al., (2010,); Doshi et al., (2010); Elbiss et al., (2013); Hammad et al., (2018); Krissi et al., (2012); Mallett et al., (2018); Ng et al.,  (2014); Po-ming & Chun-Hung, (2021);  Tanaka et al., (2014); Tinetti et al., (2018); | 22 | Bascur-Castillo et al. (2019); Bjork et al. (2014); Brown,  Rogers & Wise (2017); Buurman & Lagro-Janssen  (2013); Carroll et al., (2022); Carsughi, Santini & Lamura (2019); Chen et al. (2018); Devendorf et al.  (2020); Gore-Gorszewska (2020); Jackson et al. (2012);  Jurgensen et al. (2015); Mapp et al. (2019); Milroy,  Jacobs & Frayne, (2022); Moossdorff-Steinhauser et al.,  (2023); Pintos-Diaz et al. (2019); Roin & Nord (2015); |

|  |  | Waetjen et al., (2018); Wojtowicz et al., (2014) |  | Siddiqui et al. (2016); Tucker et al. (2019); TuiSamoa,  Heather & Kruger, (2022); Vardeman, Spiers &  Yamasalu (2022); Wagg, Kendall & Bunn (2017);  Welch, Botelho & Tennstedt (2011) |
| --- | --- | --- | --- | --- |
| Fear of examination, possible investigations,  treatments            Fear of serious disease | 17                  3 | Berger et al., (2011); Cumming et al.,  (2010); Doshi et al., (2010); Dunivan et al.,  (2015); Jarbøl et al., (2021); Krissi et al.,  (2012a); Mallett et al., (2018); Pakbaz et al.,  (2011); Po-ming & Chun-Hung, (2021);  Schreiber Pedersen et al., (2018); Smith et al., (2021); Tanaka et al., (2014); Tinetti et al., (2018); Tudor et al., (2018); Washington et al., (2013); Willis-Gray et al., (2015);  Wójtowicz et al., (2014)    Jarbøl et al., (2021); Mallett et al., (2018);  Washington et al., (2013) | 8                  4 | Brown, Rogers & Wise (2017); Chen et al. (2018);  Donaldson & Meana (2011); Grundstrom et al. (2018);  Grundstrom et al. (2020); Jackson et al. (2017);  Vardeman, Spiers & Yamasalu (2022); Wagg, Kendall &  Bunn (2017)              Bascur-Castillo et al. (2019); Ghetti et al. (2015); Welch,  Taubenberger & Tennstedt (2011); Wieslander et al.,  (2015) |
| Trivialising by  clinicians | 14                          2 | Alshammari et al., (2020); Berger et al., (2011); Cumming et al., (2010); Dunivan et al., (2015); Gambrah et al., (2022); Mallett et al., (2018); Mann et al., (2013); Po-ming & Chun-Hung, (2021); Schreiber Pedersen et al., (2018); Tinetti et al., (2018); Tudor et al., (2018); Waetjen et al., (2018);  Washington et al., (2013); Wojtowicz et al.,  (2014)                  Mann et al., (2013); Schreiber Pedersen et al., (2018) | 25                          12 | Abhyankar et al. (2019); Bjork et al. (2014);  Brown, Rogers & Wise (2017); Fileborn et al. (2017);  Gore-Gorszewska (2020); Grundstrom et al. (2018); Grundstrom et al. (2020); Hinchliff et al. (2018); Jackson et al. (2017); Jurgensen et al. (2015); Leusink et al.  (2019); Low & Tumbarello (2012); Moossdorff-  Steinhauser et al., (2023); Muller (2010); Newton et al. (2013); O’Malley, Smith & Higgins, (2021); Pakbaz et al. (2010); Roin & Nord (2015); Rutte et al. (2016); Schaller, Traeen &Lundin Kvalem (2020); Siu (2015);  Vethanayagam et al. (2017); Welch, Botelho &  Tennstedt (2011); Welch, Taubenberger & Tennstedt  (2011); Young, Fisher & Kirkman (2019);    Bascur-Castillo et al. (2019); Brown, Rogers & Wise (2017); Buurman & Lagro-Janssen (2013); Grundstrom et al. (2018); Grundstrom et al. (2020); Leusink et al. (2019); Muller (2010); Newton et al. (2013); O’Malley,  Smith & Higgins, (2021); Schaller, Traeen &Lundin |

| Perceived lack of clinician knowledge/  training |  |  |  | Kvalem (2020); Siddiqui et al. (2016); Young, Fisher & Kirkman (2019) |
| --- | --- | --- | --- | --- |
| Inconvenience | 16 | Alshenqeti et al., (2022); Dunivan et al., (2015); Gambrah et al., (2022); Hinchliff et al., (2020); Jarbol et al., (2021); Lamerton et  al., (2020); Mallett et al., (2018); Moossdorff-Steinhauser et al., (2021a,  2021b); Pakbaz et al., (2011); Schreiber  Pedersen et al., (2018); Smith et al., (2021);  Tanaka et al., (2014); Tinetti et al., (2018);  Tudor et al., (2018); Willis-Gray et al.,  (2015) | 12 | Bascur-Castillo et al. (2019); Brown, Rogers & Wise  (2017); Grundstrom et al. (2020); Hatchett et al. (2011); Jackson et al. (2017); Mapp et al. (2019); Miller, Gamnble & Barry-Kinsella, (2022); Pakbaz et al.  (2010); Siddiqui et al. (2016); Siu (2015); Vardeman,  Spiers & Yamasalu (2022);Young, Fisher & Kirkman (2019); |
| Cost of seeking healthcare | 12 | Berger et al. (2011); Dunivan et al., (2015);  Gwee & Setia, (2012); Mallett et al., (2018); Mann et al., (2013); Moossdorff-Steinhauser et al., (2021a, 2021b); Muller et al., (2010); Tanaka et al., (2014); Tinetti et al., (2018); Washington et al., (2013); Willis-Gray et al.  (2015); Wojtowicz et al., (2014) | 15 | Bascur-Castillo et al. (2019); Brown, Rogers & Wise (2017); Devendorf et al. (2020); Grundstrom et al.  (2020); Hatchett et al. (2011); Jackson et al. (2017);  Jurgensen et al. (2015); Mapp et al. (2019); Pakbaz et al.  (2010); Schaller, Traeen &Lundin Kvalem (2020);  Siddiqui et al. (2016); Siu (2015); Tucker et al. (2019);  Wieslander et al. (2015); Young, Fisher & Kirkman  (2019) |
| **FACILITATORS** |  |  |  |  |
| Increased knowledge | 3 | Krissi et al., (2012); Schreiber Pedersen et al., (2018); Tinetti et al., (2018) | 13 | Abhyankar et al. (2019); Bascur-Castillo et al. (2019);  Fileborn et al. (2017); Grundstrom et al. (2018);  Hatchett et al. (2011); Jackson et al. (2012); Low &  Tumbarello (2012); Muller (2010); Newton et al. (2013);  Pakbaz et al. (2010); Siddiqui et al. (2016); Welch,  Botelho & Tennstedt (2011); Wieslander et al. (2015) |
| Worsening symptom  bother | 9 | Doshi et al., (2010); Elbiss et al., (2013);  Hinchliff et al., (2020); Krissi et al., (2012);  Moossdorff-Steinhauser et al., (2021a.,  2021b); Pakbaz et al., (2011); Schreiber  Pedersen et al., (2018); Tinetti et al., (2018) | 7 | Abhyankar et al. (2019); Bascur-Castillo et al. (2019);  Brown, Rogers & Wise (2017); Jurgensen et al. (2015);  Pakbaz et al. (2010); Vethanayagam et al. (2017);  Welch, Taubenberger & Tennstedt (2011) |

| Fear of serious disease | 2 | Doshi et al., (2010); Schreiber Pedersen et al., (2018) | 4 | Bascur-Castillo et al. (2019); Ghetti et al. (2015); Welch, Taubenberger & Tennstedt (2011); Wieslander et al.  (2015) |
| --- | --- | --- | --- | --- |
| Stigma | 3 | Doshi et al., (2010); Hinchliff et al., (2020);  Tinetti et al., (2018) | 0 | - |
| Social support | 4 | Gwee and Setia (2012); Hinchliff et al.,  (2020); Pakbaz et al., (2011); Schreiber  Pedersen et al., (2018) | 10 | Cross et al. (2014); Donaldson & Meana (2011); Jackson et al. (2012; 2017); Jurgensen et al. (2015); Pakbaz et al.  (2010); Schaller, Traeen &Lundin Kvalem (2020);  Siddiqui et al. (2016); Vethanayagam et al. (2017);  Wang et al. (2011) |
| Supportive clinician | 0 | - | 18 | Bascur-Castillo et al. (2019) Brown, Rogers & Wise  (2017) Buurman & Lagro-Janssen (2013) Donaldson &  Meana (2011 Fileborn et al. (2017) Ghetti et al. (2015);  Grundstrom et al. (2018, 2020); Hatchett et al. (2011); Jurgensen et al. (2015); Leusink et al. (2019); Newton et al. (2013); Rutte et al. (2016); Schaller, Traeen &Lundin Kvalem (2020); Siddiqui et al. (2016); Tucker et al.  (2019); Welch, Taubenberger & Tennstedt (2011);  Young, Fisher & Kirkman (2019) |
| Assertiveness to ask for help | 0 | - | 6 | Ghetti et al. (2015); Jurgensen et al. (2015); Muller  (2010); Newton et al. (2013); Schaller, Traeen &Lundin  Kvalem (2020); Young, Fisher & Kirkman (2019) |
